# Green Ultrasound-Assisted Dispersive Liquid–Liquid Microextraction Coupled to GC–MS for Simultaneous Determination of Lipid-Peroxidation- and Maillard-Derived Carbonyl Compounds in Plant-Based Beverages

**DOI:** 10.1021/acsmeasuresciau.5c00133

**Published:** 2025-11-03

**Authors:** Jorge A. Custodio-Mendoza, Antía Villanueva, Agata Antoniewska-Krzeska, Rosa Pérez-Gregorio, Elena Martínez-Carballo, María Llompart, Antonia María Carro-Díaz

**Affiliations:** † Instituto de Agroecoloxía e Alimentación (IAA) − Food and Health Omics, 73032 Universidade de Vigo, Campus Auga, 32004 Ourense, Spain; ‡ Department of Analytical Chemistry, Nutrition and Food Science, Faculty of Chemistry, 16780 Universidade de Santiago de Compostela (USC), 15782 Santiago de Compostela, Spain; § Institute of Human Nutrition Sciences, Faculty of Human Nutrition, Warsaw University of Life Sciences, Nowoursynowska 159c, 02-776 Warsaw, Poland; ∥ CRETUS − Department of Analytical Chemistry, Nutrition and Food, USC, 15782 Santiago de Compostela, Spain; ⊥ Health Research Institute of Santiago de Compostela (IDIS), USC, 15782 Santiago de Compostela, Spain; # Instituto de Materiais (iMATUS), USC, 15782 Santiago de Compostela, Spain

**Keywords:** Food safety monitoring, Furfural, GC−MS, Green analytical chemistry, Malondialdehyde, Plant-based beverages, DLLME

## Abstract

Carbonyl compounds generated through lipid peroxidation and Maillard-type reactions are relevant markers of food quality and safety due to their potential toxicity and prevalence in processed plant-based beverages. This work presents the development and validation of a green, ultrasound-assisted dispersive liquid–liquid microextraction (UA-DLLME) method using sustainable extractant (isobutyl acetate) and dispersant (isopropanol) solvents for the simultaneous determination of 12 carbonyl compounds across multiple chemical families (aldehydes, ketones, furans, and dicarbonyls). The method integrates in situ derivatization with *O*-(2,3,4,5,6-pentafluorobenzyl) hydroxylamine hydrochloride and gas chromatography–mass spectrometry (GC–MS) analysis. Method optimization was performed using a multivariable approach using an asymmetrical 2^6^·3^1^ screening design and a central composite design for response surface methodology. Analytical performance was validated according to Food and Drug Administration guidelines, achieving great linearity (*r* ^2^ ≥ 0.9991), accuracy (90–107% recovery), precision (<7.5% RSD) and detection limits suitable for trace-level analysis in complex matrixes. The approach was applied to 51 commercial plant-based beverage samples (almond, soy, oat, rice, coconut, seed and mixed formulations). Greenness assessment was performed using AGREEprep (0.69/1) and BAGI (77.5/100) metrics, confirming the method’s alignment with green chemistry principles. This workflow provides a reliable and sustainable strategy for routine monitoring of carbonyls in food systems, with relevance to both quality control and exposure assessment.

## Graphical abstract

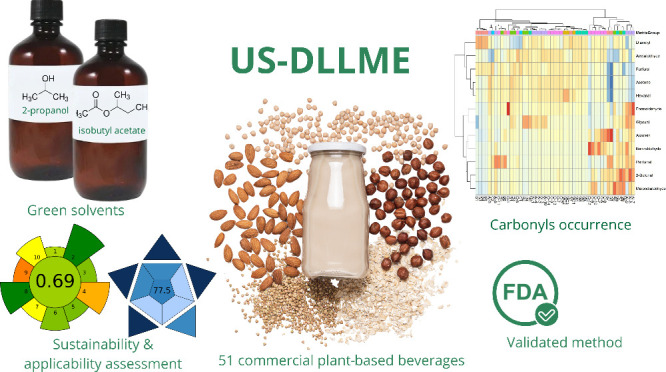

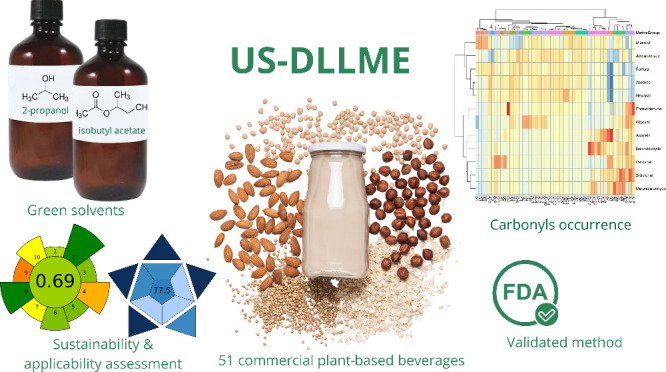

Plant-based beverages (PBBs) are gaining growing importance in the food industry as alternatives to conventional dairy products, driven by consumer demand for more sustainable and health-oriented diets.^1^ While dairy products remain nutritionally complete, PBBs are recognized for their content of bioactive phytochemicals, including antioxidants and anti-inflammatory agents, which may support cardiovascular and metabolic health.^2^ Moreover, they stand as an alternative for individuals with lactose intolerance, milk protein allergies, or those following low-cholesterol diets.^3,4^ In Spain, the consumption of PBBs has steadily increased in recent years. According to data from the Ministry of Agriculture, Fisheries and Food,^5^ national consumption grew from approximately 226 million liters in 2017 to over 289 million liters by 2023. Notably, organic PBBs labeled as *bio* in accordance with European food labeling regulations (Regulation (EU) 2018/848 ^6^) began to be recorded as a separate category in 2020, reaching over 31 million liters in their first year and maintaining a significant share of the market in subsequent years. This continued growth underscores Spain’s leading role in the European plant-based beverage sector, with national trends reflecting broader EU dietary shifts that position these products as functional, health-oriented alternatives.^4^

PBBs are typically produced from raw materials such as cereals, nuts, legumes, or seeds and undergo multistep processing to mimic the sensory and nutritional characteristics of dairy.^7^ Standard operations include soaking, grinding, pasteurization, and, in many cases, Ultra-High Temperature (UHT, 135–150 °C for 2–4 s) treatment, while additional health treatments, such as roasting, are often employed to enhance flavor.^7–9^ However, these thermal processes, together with extended storage, can compromise chemical stability by promoting nonenzymatic reactions such as the Maillard reaction and lipid peroxidation. These pathways are particularly relevant, as they contribute to the formation of reactive carbonyl species of toxicological concern.^9–11^ Compounds encompassing saturated and unsaturated aldehydes, ketones, dicarbonyls, and furan derivatives (Table 1) are generated as byproducts of sugar degradation and lipid oxidation and have been associated with cytotoxic and genotoxic effects at elevated concentrations.^11–19^ International agencies such as the International Agency for Research on Cancer (IARC) have classified several of these compounds based on the strength of evidence regarding their carcinogenicity, using a system that includes: Group 1 (carcinogenic to humans), Group 2A (probably carcinogenic to humans), Group 2B (possibly carcinogenic to humans), and Group 3 (not classifiable as to its carcinogenicity to humans).^12^

**Table 1:** Toxicological Reference Values and the Regulatory Classification of Selected Carbonyl Compounds^a^

Carbonyl Compounds	IARC Classification^12^	Reference Value (μg/kg bw/day)
Formaldehyde	Group 1	TDI: 150 ^13^
Acetaldehyde	Group 2B	AI: 185 ^14^
Hexanal	–	ADI: 780 ^15^
Diacetyl	–	ADI: 900 ^16^
Malondialdehyde	Group 3	TTC: 30 ^17^
Glyoxal	–	TDI: 200 ^16^
Acrolein	Group 2A	TDI: 7.5 ^18^
Crotonaldehyde	Group 2B	–
Furfural	Group 3	TDI: 500 ^19^

a IARC, International Agency for Research on Cancer; bw, body weight; TDI, tolerable daily intake; AI, acceptable intake reported at μg/day; ADI, acceptable daily intake; TCC, threshold of toxicological concern set by The International Programme on Chemical Safety (IPCS).

In parallel, international regulatory agencies have established various toxicological reference values to assess and limit chronic dietary exposure to these compounds.^13–19^ The tolerable daily intake (TDI) is defined by the European Food Safety Authority (EFSA) as the estimated amount of a substance that can be ingested daily over a lifetime without appreciable health risk.^13,16–19^ The acceptable daily intake (ADI) is established by the Joint Food and Agricultural Organization (FAO) and the World Health Organization (WHO) Expert Committee on Food Additives (JECFA) for substances intentionally added to food, such as additives or processing aids.^15,16^ In cases where toxicological data are scarce, the threshold of toxicological concern (TTC) approach, developed by the WHO, provides a risk assessment tool based on structural and exposure considerations.^17^ The presence of these carbonyls, even at trace levels, underscores the importance of reliable and sensitive analytical methods for their quantification in PBBs.^11^

The analytical determination of carbonyl compounds in food matrixes has been extensively addressed through a variety of instrumental approaches, which include both spectroscopic and chromatographic techniques.^11^ Among the former, assays such as the thiobarbituric acid reactive substances test have been widely used to estimate malondialdehyde (MDA) and other lipid oxidation products.^20^ However, these methods often lack selectivity and are prone to interferences in complex matrixes.^11,20^ For compound-specific identification and quantification, chromatographic techniques, particularly gas chromatography (GC) and high-performance liquid chromatography (HPLC), have become the gold standard due to their sensitivity, selectivity, and compatibility with different detection systems including ultraviolet (UV), fluorescence (FLD) and mass spectrometry (MS) detection.

Sample preparation remains a critical step in the analytical workflow, with increasing emphasis on miniaturized and environmentally friendly techniques.^11,21,22^ Microextraction approaches such as solid-phase microextraction (SPME), gas-diffusion microextraction (GDME), and dispersive liquid–liquid microextraction (DLLME) have gained popularity due to their minimal solvent use, reduced sample size and waste generation, high efficiency, potential for in situ derivatization during extraction, and suitability for automation.^11,23–25^ Nevertheless, the low volatility and chemical reactivity of many carbonyl compounds often require prior derivatization to enhance the stability and detectability. A broad range of derivatizing agents have been employed, including hydrazine, diamine, and hydroxylamine derivatives.^11^ Among them, 2,4-dinitrophenylhydrazine (DNPH) remains the most extensively used, particularly in HPLC–UV applications. For example, DNPH has been applied for the determination of furfural (FUR) and acetaldehyde (ACE) in beer using GDME coupled with HPLC–UV (Gonçalves et al., 2010),^23^ as well as for the simultaneous GC–MS determination of malondialdehyde (MDA), acrolein (ACRL), and other aldehydes in edible oils using GDME and DLLME (Custodio-Mendoza et al., 2020).^24^ Among non-hydrazine-based agents, hydroxylamine derivatives have become particularly relevant for GC–MS. The most widely employed is *O*-(2,3,4,5,6-pentafluorobenzyl) hydroxylamine (PFBHA), which forms thermally stable oxime derivatives with a wide variety of carbonyls. This reagent has been successfully used for the quantification of more than 40 carbonyls, including FUR, ACRL, and glyoxal (GO), in beer and port wines via SPME–GC–MS (Moreira et al., 2019).^25^ DLLME, in particular, stands as a promising technique for aqueous food, due to its compatibility with different detection systems including HPLC and GC, rapidity, and further low organic solvent consumption.^11^ However, it has the disadvantage of regularly using halogenated or otherwise toxic disperser and extraction solvents.^11,24^ In addition, thermostated agitation is often needed to achieve specific conditions for an effective extraction, particularly when simultaneous derivatization is involved. To address these limitations, techniques such as microwave (MW) and ultrasound (US) are commonly employed since their physical features (e.g., cavitation phenomena in US) can further enhance extraction efficiency. Nevertheless, their application may result in higher energy consumption, which conflicts with the principles of green analytical chemistry. This highlights the need for safer and more sustainable alternatives.^11,24–26^

In this study, we developed and validated an ultrasound-assisted dispersive liquid—liquid microextraction method using sustainable solvents. The in situ PFBHA derivatization allowed for the simultaneous determination of 12 carbonyl compounds in PBBs by GC-MS. This green method was thoroughly validated according to Food and Drug Administration (FDA) guidelines, ensuring analytical reliability and accuracy. Moreover, we assessed the environmental and practical performance of this method using novel metrics for sustainability and applicability, thereby contributing novel insights into its feasibility for use in the food industry. This method was used to assess the occurrence of these carbonyls in 51 PBBs further proving their viability for quality control in these foods. This work aims to bridge the gap between experimental research and practical applications by offering a robust, reproducible, and greener extraction protocol that supports industrial implementation and standardization efforts.

## Materials and Methods

### Chemicals

All reagents used were of analytical grade (≥90% purity) and employed without further purification unless otherwise indicated. 2,4-Dinitrophenylhydrazine (DNPH, CAS 119-26-6), acetonitrile (ACN, CAS 75-05-8), isooctane (CAS 540-84-1), diethyl carbonate (DEC, CAS 105-58-8), dimethyl carbonate (DMC, CAS 616-38-6), ethanol (EtOH, CAS 64-17-5), hydroxylamine hydrochloride (NH_2_OH, CAS 5470-11-1), isobutyl acetate (IBA, CAS 110-19-0), methanol (MeOH, CAS 67-56-1), methoxylamine hydrochloride (MOX, CAS 593-56-6), PFBHA (CAS 57982-78-2), and *O*-(*tert*-butyldimethylsilyl)­hydroxylamine (TBS-ONH_2_, CAS 99705-40-3) were all acquired from Merck (Darmstadt, Germany). 2-Propanol (IPA, CAS 67-63-0) and hydrochloric acid (CAS 7647-01-0) were obtained from VWR Chemicals (Radnor, PA, USA). Analytical standards of ACE (CAS 75-07-0), ACRL (CAS 107-02-8), benzaldehyde (PhCHO, CAS 100-52-7), crotonaldehyde (2=C4AL, CAS 4170-30-3), diacetyl (DA, CAS 431-03-8), formaldehyde (FCHO, 37%, CAS 50-00-0), FUR (CAS 98-01-1), GO (40%, CAS 107-22-2), hexanal (HEX, CAS 66-25-1), MDA (CAS 542-78-9), and pentanal (C5AL, CAS 110-62-3) were supplied by Merck, acetone (ACO, CAS 67-64-1) was provided by VMR Chemicals, and deuterated species of acetone (ACO-*d*_6_, CAS 666-52-4) and hexanal (HEX-*d*_12_, CAS 25358-85-4) were also from Merck and used as internal standards (IS).

Ultrapure water was obtained from a Millipore purification system (Merck, Darmstadt, Germany). Stock solutions of each analyte (100–300 μg/mL) were prepared in MeOH and stored in amber glass vials (4 mL) at −18 °C. Working solutions containing all analytes (2 μg/mL) and internal standards (100 μg/mL) were freshly prepared in ultrapure water before each analysis. Derivatization solutions were prepared as follows. PFBHA was dissolved at 4 mM in 0.1 M aqueous HCl and stored at 4 °C for a maximum of 3 days. DNPH was prepared at 2 mg/mL in acidified ACN (2 M HCl). MOX was dissolved at 20 mM in 1 M phosphate buffer (pH 6.0). TBS-ONH_2_ was prepared at 5 mM concentration in acetonitrile. Hydroxylamine hydrochloride (NH_2_OH) was dissolved in Milli-Q water at 50 mM and adjusted to pH 7.0 by using sodium hydroxide. All solutions were stored at 4 °C and used within 1 week.

### Sample Collection and Storage

A total of 51 commercial plant-based beverage samples were collected from local supermarkets and health food stores in Santiago de Compostela, Spain. The selection encompassed a diverse range of formulations and brands, including beverages based on almond (*n* = 10), coconut (*n* = 2), oat (*n* = 14), rice (*n* = 7), soy (*n* = 8), minor crops (*n* = 3) including rye-, canary-seed-, and tiger-nut-based, and mixed formulations (*n* = 7) combining different raw materials: oat–hazelnut–nut, rice–almond, soy–pea, soy–oat–coconut, rice–hazelnut, oat–nut, and rice–coconut. All samples were stored in their original packaging, protected from light, and kept at room temperature prior to analysis. After opening, aliquots were taken for analysis, and each sample was flushed with nitrogen gas to remove residual air, sealed with parafilm to prevent oxidation, and stored at −18 °C.

A nonroasted almond-based beverage was selected as the sample blank, and quality control substances (QCs) were freshly prepared by spiking the sample blank with a known concentration of analytes at 200 ng/mL (QC1), 400 ng/mL (QC2), and 600 ng/mL (QC3) and used in the validation studies.

### Ultrasound-Assisted Dispersive Liquid–Liquid Microextraction

A schematic representation of the optimized method is provided in Figure 1. Briefly, 0.5 mL of sample was transferred to a 5 mL Eppendorf tube, followed by the addition of 1 mL of IPA (disperser solvent) and 1 mL of IBA (exclusively for defatting). The mixture was vortexed for 1 min and centrifuged at 3000 rpm for 5 min to achieve phase separation. Proteins precipitated to form a pellet at the bottom, while lipids were retained in the upper organic layer, which was discarded. The intermediate IPA phase, containing the analytes, was carefully transferred to a clean 2 mL glass vial. At this stage, a fresh aliquot of IBA (90 μL) was added as the extraction solvent. In parallel, the derivatization solution was prepared in a conical glass tube by combining 0.5 mL of PFBHA (4 mM in 0.1 M HCl) and 50 μL of IS solution and brought to a final volume of 5 mL with ultrapure water. The resulting solution presented a pH of 2.0, which is a condition required to ensure the complete derivatization of the target carbonyl compounds. The isopropanol/isooctane extract was then rapidly added to the derivatization solution using a Pasteur pipet. The resulting mixture was placed in an ultrasonic bath at 40 °C for 20 min to allow for simultaneous derivatization and extraction. After sonication, samples were centrifuged at 3500 rpm for 5 min to separate phases. The upper organic layer, containing the oxime derivatives, was carefully collected with a micro syringe and transferred to chromatographic vials with glass inserts for subsequent GC–MS analysis.

**Figure 1:**
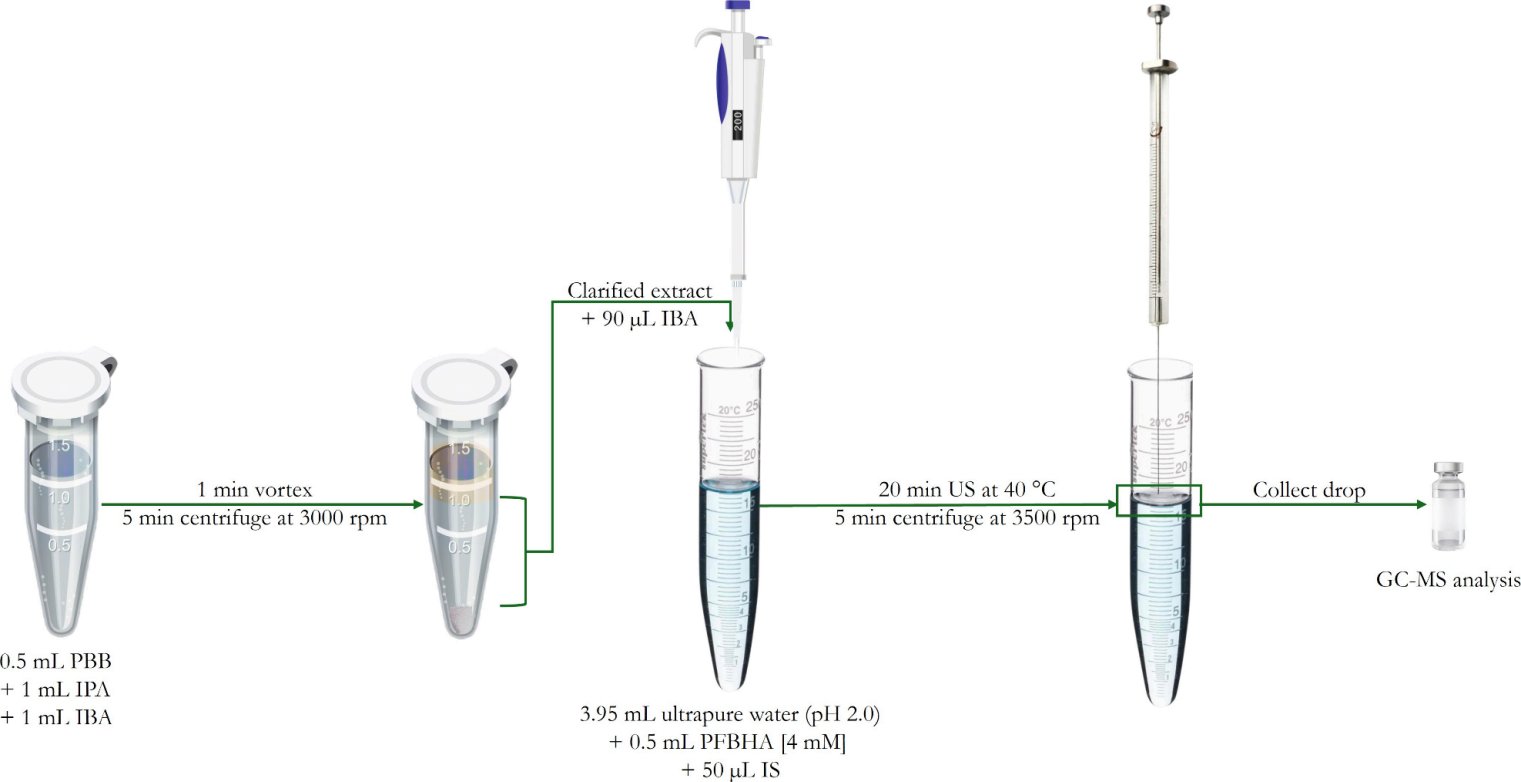
Scheme of the US-DLLME of carbonyl compounds from plant-based beverages (PBBs). IPA, isopropanol; IBA, isobutyl acetate; PFBHA, *O*-(2,3,4,5,6-pentafluorobenzyl) hydroxylamine hydrochloride; IS, internal standard; US, ultrasound.

### Gas Chromatography–Mass Spectrometry

Gas chromatography–mass spectrometry (GC–MS) analyses were performed using an Agilent 7890B gas chromatograph coupled to a 5977B single quadrupole mass selective detector (Agilent Technologies, Santa Clara, CA, USA). Chromatographic separation was achieved using an HP-5MS capillary column (30 m × 0.25 mm i.d., 0.25 μm film thickness; Agilent J&W Scientific). Helium (99.999%) was used as the carrier gas at a constant flow rate of 1.5 mL/min. The injector was operated in splitless mode at 245 °C, equipped with an ultrainert double taper liner. The oven temperature program was as follows: initial temperature 100 °C (held for 2.5 min), increased at 100 °C/min to 230 °C (held for 3.3 min), then ramped at 35 °C/min to 280 °C (held for 4 min), resulting in a total runtime of 12.5 min. The interface temperature was maintained at 280 °C. The mass spectrometer operated under electron impact ionization (EI) at 70 eV in positive mode. The ion source temperature was set at 250 °C and the quadrupole at 120 °C. Detection was performed initially in full scan mode within 50–500 *m*/*z* to identify the specific ions for each derivatized carbonyl compound to then perform the analysis in selected ion monitoring (SIM) mode to enhance sensitivity and specificity for the analytes. Retention times and target ions for each analyte are detailed in the Analytical Figures of Merit section.

### Analytical Validation

Analytical performance was evaluated for the 12 targeted carbonyl compounds using analytical standards and a nonroasted almond-based beverage as blank sample. Method validation was carried out in accordance with Food and Drug Administration (FDA) guidelines for the validation of analytical methods.^27,28^ The parameters assessed included specificity, linearity, sensitivity (LOD and LOQ), accuracy, and precision. Specificity was verified by confirming the absence of interfering signals at the retention times of each analyte in the matrix. The method’s capacity to differentiate the derivatized compounds in a complex plant-based matrix was ensured through selective ion monitoring (SIM), using quantifier and qualifier ions detailed in the Analytical Figures of Merit section. Linearity was evaluated by constructing standard addition with internal standard calibration curves with at least six concentration levels with three replications per level covering the full analytical range of interest for each compound. The limits of detection (LOD) and quantification (LOQ) were estimated based on the standard deviation of the blank signal: LOD as 3.3 times and LOQ as 10 times the standard deviation were added to the mean blank signal. Accuracy was assessed by calculating recoveries (%) from quality control samples spiked at three levels, in quintuplicate, representative of the working range. Precision was evaluated as intraday and interday repeatability, with results expressed as relative standard deviation (%RSD) from five replicate measurements (*n* = 5). The %RSD values remained within the acceptable thresholds for bioanalytical methods, confirming the robustness and reproducibility of the procedure. The matrix effect was evaluated by comparing the slope of the standard addition calibration plots with those of the external calibration (in absence of sample) plots. Ratios between 80 and 120% are considered indicative of the absence of the matrix effect.

### Statistics and Multivariant Optimization

All experimental design and statistical analyses were performed using NemrodW software (LPRAI, Marseille, France), following a two-step optimization strategy. First, a univariate preselection was conducted to identify promising conditions for key parameters such as derivatization temperature, aqueous phase volume, type and volume of extractant and dispersant, and derivatizing agent. These preliminary results provided the foundation for a multivariate optimization approach that enabled the simultaneous evaluation of multiple factors. An asymmetric screening design (2^6^3^1^) comprising 12 experiments was used to evaluate seven variables at different levels: derivatization temperature (40 °C, 60 °C), aqueous phase volume (3 mL, 5 mL), extractant type (IBA, DMC), extractant volume (60 μL, 90 μL), dispersant type (IPA, ACN), dispersant volume (0.7 mL, 1.3 mL), and derivatizing agent (DNPH, PFBHA, TBS-ONH_2_). This screening revealed the most influential variables for extraction efficiency based on normalized peak areas of the target analytes. Following the screening, a central composite design (CDD) was implemented to fine-tune the two most critical parameters, the extractant and dispersant volumes. The design included two factors with five levels each and three replicates at the center point, resulting in a total of 11 experiments. Desirability functions were then applied to determine the optimal conditions.

In addition, occurrence data of 12 carbonyl compounds across the set of 51 PBBs were subjected to descriptive and inferential statistics using R software (R Core Team, version 4.5.0). Occurrence frequencies, medians, interquartile ranges, and ranges were calculated by a matrix group. Group differences were assessed through Kruskal–Wallis tests, followed by Dunn’s post hoc comparisons with Benjamini–Hochberg correction, and nonparametric effect sizes were estimated. Multivariate analyses were performed on log-transformed relative concentration profiles, including principal component analysis (PCA) to explore clustering tendencies, hierarchical clustering with heatmaps to visualize co-occurrence patterns, and permutational multivariate analysis of variance (PERMANOVA) to test overall differences among matrixes. Figures and tables were generated using the packages ggplot2, FactoMineR, factoextra, pheatmap, and vegan.

### Sustainability and Applicability Metrics

The environmental impact of the sample preparation strategy was assessed using the AGREEprep tool, freely available software specifically developed to evaluate the greenness of sample preparation procedures. The evaluation is based on ten criteria that reflect the principles of green analytical chemistry, each scored from 0 (least sustainable) to 1 (most sustainable). These individual scores are combined using default weighting factors to yield an overall score on a 0–1 scale, where values closer to 1 represent better environmental performance. This assessment made it possible to identify both strengths and opportunities for improving the sustainability of protocol.^29,30^ To complement this evaluation, the Blue Applicability Grade Index (BAGI) was applied to assess the overall practicality of the developed method. This tool, accessible via an online platform (https://bagi-index.anvil.app/), examines ten key parameters grouped into two main domains: analytical determination and sample handling. Each criterion is scored on a scale from 2.5 to 10 points using a color-coded system, where dark blue denotes high applicability and white indicates limited suitability. The BAGI tool provided a structured overview of the method’s real-world applicability, highlighting its feasibility for routine implementation and areas where further refinement could enhance performance.^31^

## Results and Discussion

### Optimization of the Extraction and Derivatization Procedures

The main objective of this research was to establish optimal conditions for the simultaneous extraction and derivatization of carbonyls from PBBs with a reduced environmental impact compared to current methods using DLLME. Parameters that potentially affect the effectiveness of DLLME include the type and amount of dispersant and extractant solvent, aqueous phase volume, time, and temperature, and the derivative reagent, among others. In this study, four hydroxylamines were evaluated as derivatization reagents encompassing TBS-ONH_2_, PFBHA, NH_2_OH, and MOX based on their ability to form oximes with carbonyl compounds, as an alternative to the most used DNPH.^32^ Oximation converts the carbonyl group into a more stable and less volatile oxime, which improves detectability and chromatographic performance in GC–MS analysis.^33^ Individual derivatizations of target analytes were performed using the US-DLLME procedure previously published, each in triplicate.^32^ Extracted ion chromatograms (EICs) of the precursor ion of each corresponding carbonyl oxime are presented in Figures S1–S4 and Table S1 of the Supporting Information. As shown in Table S1 and Figures S1 and S2, only TBS-ONH_2_ and PFBHA produced stable and clearly distinguishable signals, with precursor ions larger than 100 Da, while NH_2_OH and MOX produced oximes of relatively low molecular weight with reduced sensitivity. This higher molecular mass is advantageous in GC–MS because it minimizes spectral interference and enhances selectivity, making these reagents suitable for the scope of this study. Therefore, they were selected for further optimization together with other experimental parameters by an experimental screening design. The formation of oximes requires acidic conditions,^33^ typically around pH 2. Most applications employ hydrochloric acid for this purpose. In this work, citric acid and phosphoric acid were also tested, adjusting the pH of the solution to 2, each in triplicate. The results, expressed as relative chromatographic areas, are shown in Figure 2.

**Figure 2:**
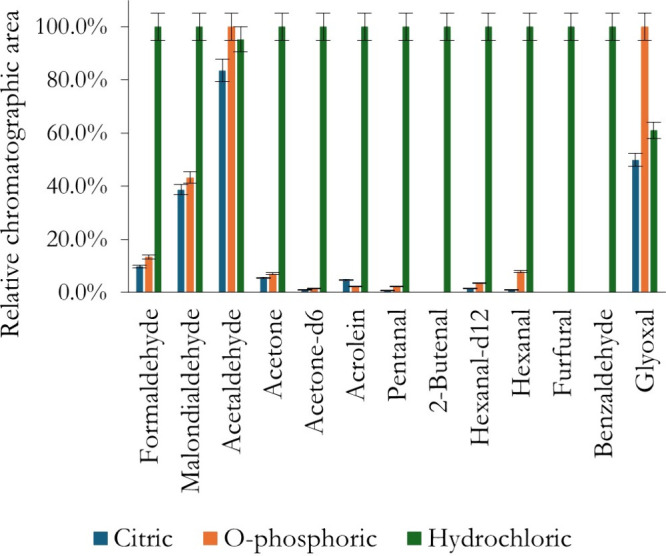
Study of the effect of the type of acid on the efficiency of the US-DLLME of carbonyls from PBBs.

Despite the identical pH readings, HCl provided consistently higher relative chromatographic areas for most of the analytes, except for ACE and GO; similar and higher areas were obtained when using *O*-phosphoric acid. This effect can be attributed to the influence of buffer anions on nucleophilicity: phosphate and citrate anions promote ion pairing with the protonated hydroxylamine, lowering the fraction of reactive nucleophile available, while chloride is weakly coordinating and interferes less with reaction.^34^ Similar buffer effects on oxime and hydrazone formation have been reported in the literature, where phosphate buffers significantly slow reaction rates compared to chloride- or amine-based catalytic buffers, even at the same pH;^33,34^ thus HCl was kept for further tests. Despite the use of HCl, the concentration applied here (4 mM) is much lower than in conventional methods, which often employ up to 2 M, thereby significantly reducing the environmental impact of the methodology.^32^ Reaction temperature is also a crucial factor directly influencing the kinetics of oxime formation. Two reaction temperatures were studied, 40 and 60 °C, based on the reported literature (Figure 3).

**Figure 3:**
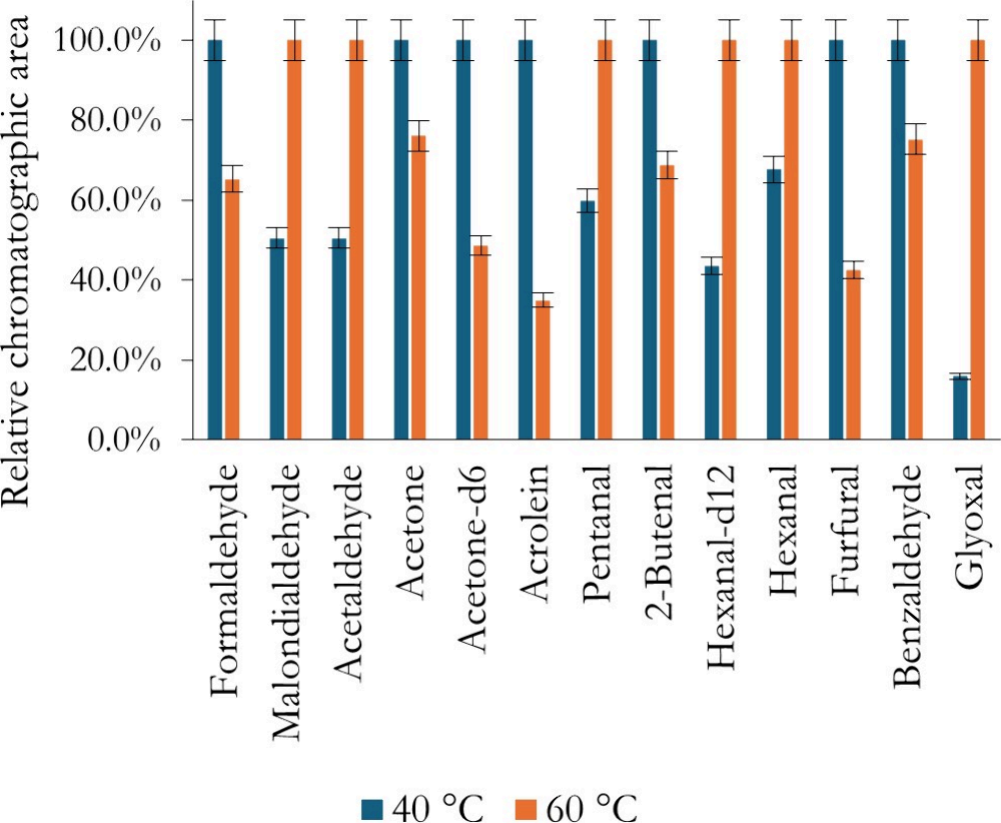
Study of the effect of the temperature on the efficiency of the US-DLLME of carbonyls from PBBs.

As observed, there is no universal optimal temperature. For more volatile analytes, such as furfural, higher temperatures may promote partitioning into the headspace, leading to signal loss. In contrast, less volatile compounds such as malondialdehyde benefited from higher temperatures, yielding stronger chromatographic responses due to accelerated reaction kinetics. Therefore, the temperature was included as a factor in the multivariate optimization. Regarding the extractant solvent, four “green” solvents were tested: DEC, DMC, IBA, and isooctane. These solvents were selected for their low toxicity, biodegradability, and favorable environmental profiles, which align with the principles of green analytical chemistry.^35,36^

Their extraction capability toward the formed oximes was tested in triplicate (Figure 4). For these experiments, ACN was used as a dispersant to compare with previous methodologies. The highest chromatographic signals were obtained with DMC and IBA for most of the compounds. This can be attributed to their physicochemical properties: DMC has moderate polarity and low viscosity, facilitating efficient mass transfer, while IBA combines hydrophobicity with good solvating power for semipolar oximes, thus enhancing partitioning into the organic phase.^37^ On this basis, DMC and IBA were retained for further testing in the multivariate optimization. These solvents were also tested as defatting agents in the preliminary cleanup, showing no significant differences compared to the commonly used, more toxic hexane (data not shown). For coherence and reduced operational costs, the same solvent that performed best as extractant was also employed as a defatting agent. Due to their immiscibility and lower density than water, these solvents formed a floating phase. After aqueous phase removal, interfacial effects such as surface tension promoted the aggregation of this phase into a stable “drop”. Four solvents were tested as dispersants: ACN, IPA, MeOH, and EtOH. Their ability to promote protein precipitation from the sample matrix was preliminarily evaluated (Figure S5). ACN and IPA produced a compact and well-separated protein pellet, whereas methanol and ethanol gave diffuse precipitation. This agrees with previous studies reporting that ACN achieves higher protein removal efficiency than EtOH or MeOH due to their lower dielectric constants and stronger dehydration effects.^38,39^ Consequently, ACN and IPA were selected for inclusion in the multivariate optimization.

**Figure 4:**
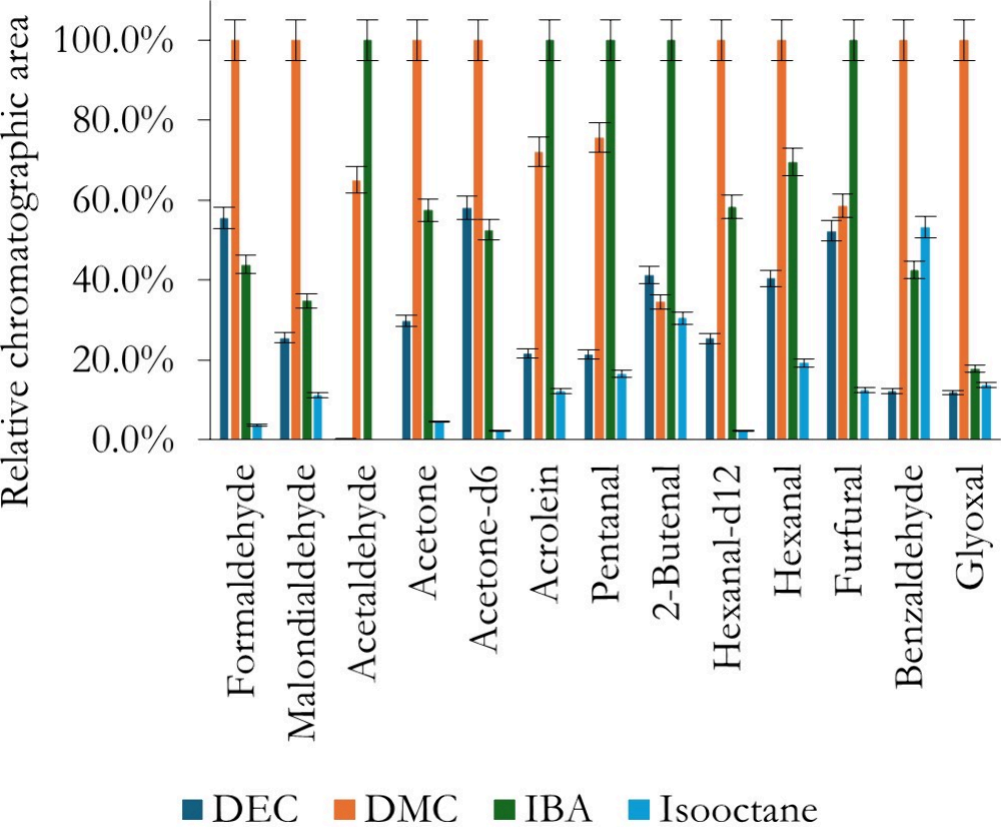
Evaluation of the effectiveness of green solvents as extractants in the US-DLLME of carbonyls from PBBs. ACN was used as a dispersant.

### Factor Screening and Response Surface Optimization

Optimization of the DLLME–derivatization procedure began with a screening stage to evaluate the influence of seven variables: derivatization temperature, aqueous phase volume, extractant type and volume, dispersant type and volume, and derivatizing agent. An asymmetric 2^3^6^1^ design (12 experiments) was applied, and results were interpreted using delta weight showing limits of statistical significance (dotted vertical lines) and total-effect plots (Figure 5 for DA and FCHO; Figures S6 and S7 for other analytes).

**Figure 5:**
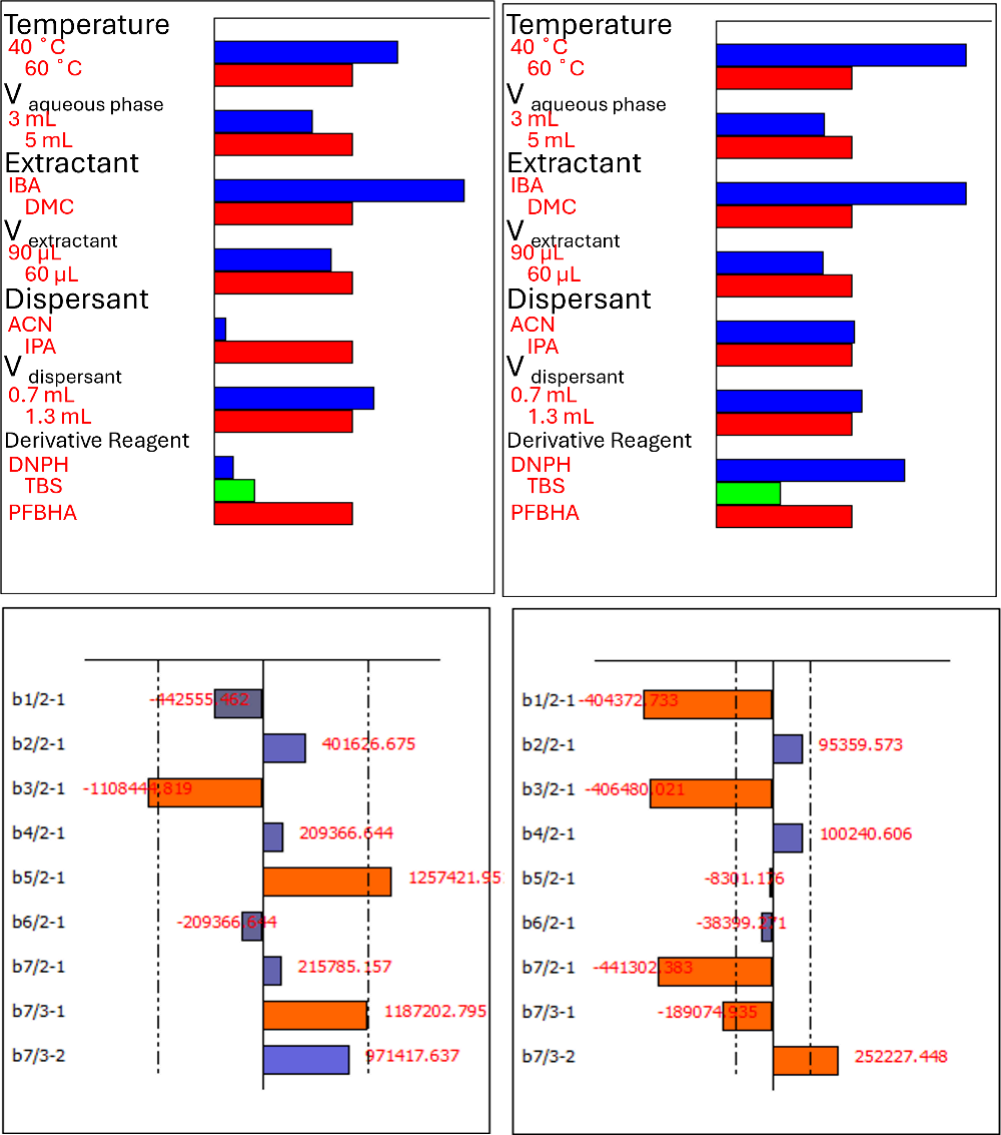
Delta weight (below) and total effect plots (above) of the asymmetrical screening design for diacetyl (A, C) and formaldehyde (B, D).

As shown, the temperature played a decisive role. At 40 °C, the signal improved notably for FCHO and C5AL, while ACO showed a slight advantage at 60 °C. Because most analytes benefited from the lower temperature, 40 °C was selected to improve simultaneous performance while avoiding excessive heating, which could otherwise compromise analyte stability. Aqueous phase volume was another critical factor. Increasing the volume from 3 to 5 mL enhanced reproducibility and ensured sufficient partitioning for all analytes. Thus, 5 mL was chosen as the working condition. The type of extractant had a clear effect. IBA consistently outperformed DMC, particularly FCHO, DA, and C5AL, confirming its superior extraction capability in the DLLME format. Similarly, dispersant type was a determinant: IPA produced stronger responses than ACN, with statistically significant improvements for DA and HEXd12. These observations led to the selection of IBA and IPA as the optimal extractant–dispersant pair. The derivatizing agent strongly influenced the sensitivity and selectivity.

Although DNPH gave high responses for FCHO and ACO and TBS-ONH_2_ performed best for 2=C4AL, ACRL, and HEX, PFBHA provided overall higher chromatographic responses and improved peak resolution across the analyte set. For this reason, PFBHA was selected.

Extractant and dispersant volumes did not show clear trends at this stage and were therefore retained as factors for refinement in subsequent RSM optimization. To fine-tune solvent volumes, a CCD (11 experiments) was conducted, focusing on extractant (50–90 μL) and dispersant (0.7–1.0 mL), while fixing other parameters at the optimal values from the screening (40 °C, 5 mL aqueous phase, IBA as extractant, IPA as dispersant, PFBHA as derivatizing agent, no salt). Global desirability analysis (Figure 6 for overall desirability; Figure S8 for individual surface responses) consistently pointed to the same optimum: 90 μL of extractant combined with 1.0 mL of dispersant.

**Figure 6:**
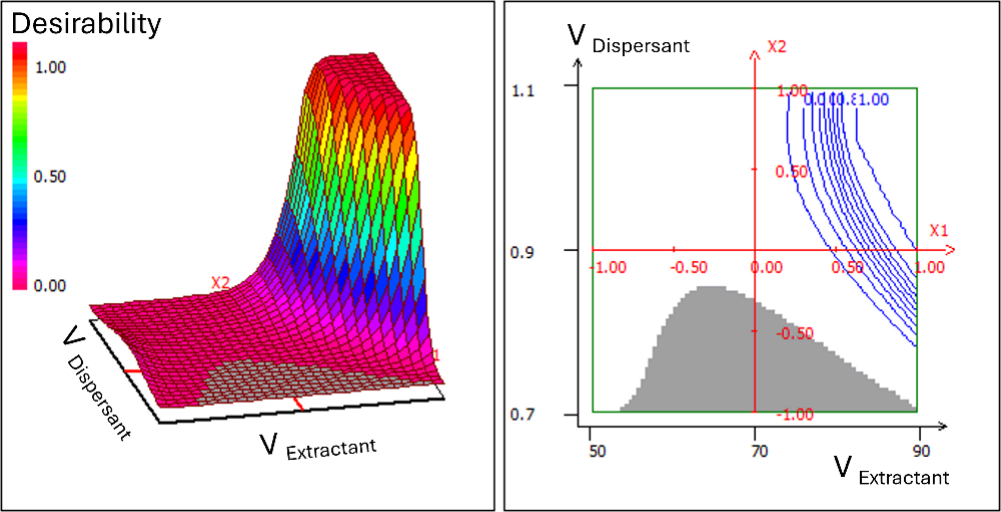
Global desirability surface obtained from the RSM optimization of DLLME parameters. 3D plot (left), 2D plot (right).

The response surfaces revealed a narrow, well-defined optimum, confirming the robustness and reproducibility of these conditions. Finally, under these optimal conditions, the salting-out effect was assessed by adding NaCl to the system. In the case of PFBHA–IBA, 20% NaCl increased the organic phase volume by coextracting dispersant, which in turn reduced the enrichment factor. As no significant improvement in extraction efficiency was achieved, salt was discarded.

### Analytical Figures of Merit

Analytical features of the optimized US-DLLME were assessed in accordance with FDA guidelines, and the results, including sample determination limits, linearity, accuracy, and precision, are summarized in Table 2.

**Table 2:** Analytical Features of the Ultrasound-Assisted Dispersive Liquid–Liquid Microextraction of Carbonyls from Plant-Based Beverages^a^

								Intraday precision			Interday precision			Accuracy		
								% RSD (*n* = 5)			% RSD (*n* = 5)			% Recovery (*n* = 5)		
Analyte	RT (min)	Quant (*m*/*z*)	Qual (*m*/*z*)		LOD (ng/mL)	LOQ (ng/mL)	*r* ^2^	QC1	QC2	QC3	QC1	QC2	QC3	QC1	QC2	QC3
Acetaldehyde	3.28	237	223	233	45.1	224.05	0.9995	0.90	0.08	0.37	2.73	0.42	5.00	90.0	99.4	100.9
Formaldehyde	3.61	267	180	251	133.3	400.8	0.9999	0.06	0.06	0.03	5.78	4.49	7.55	100.1	100.8	100.5
2-Butenal	3.92	265	266	264	8.5	39.3	0.9995	0.57	0.88	0.35	2.51	1.64	1.28	103.9	102.0	102.1
Diacetyl	3.94	264	281	282	201.3	501.3	0.9999	1.17	0.95	1.30	2.59	3.35	1.60	107.1	104.5	101.7
Acetone	4.01	252	239	240	137.2	319.1	0.9995	0.79	1.04	1.60	2.73	3.01	3.04	91.6	97.3	93.5
Pentanal	4.01	281	264	253	42.7	204.4	0.9993	1.59	0.26	0.93	2.79	1.24	2.40	97.7	102.9	101.1
Acrolein	4.09	266	221	267	265.8	509.9	0.9999	0.62	0.65	0.59	1.87	1.26	1.75	97.1	98.7	98.9
Furfural	4.24	257	243	244	64.8	343.4	0.9999	1.50	0.63	1.08	2.95	2.72	2.45	95.8	96.6	98.0
Hexanal	4.25	294	295	278	72.9	218.4	0.9992	0.72	1.11	0.48	3.71	3.05	2.93	104.6	100.3	101.4
Benzaldehyde	4.80	301	300	302	13.9	38.5	0.9998	0.36	0.48	1.36	1.24	2.90	2.55	98.9	100.2	94.2
Glyoxal	5.56	251	418	448	33.9	116.1	0.9998	1.58	1.46	0.45	2.37	2.37	2.26	103.2	98.5	99.0
Malondialdehyde	5.95	250	279	181	30.8	443.6	0.9991	1.59	1.04	1.14	3.37	1.60	2.94	105.6	104.3	104.6
Acetone-*d*_6_ (IS)	3.25	259	229	241												
Hexanal-*d*_12_ (IS)	4.23	307	289	290												

a RT, retention time; Quant, quantitative ion; Qual, qualitative ion; LOD, limit of detection; LOQ, limit of quantification; RSD, relative standard deviation; QC1, 200 ng/mL; QC2, 400 ng/mL; QC3, 600 ng/mL; IS, internal standard; *n*, numbers of replicates per studied level.

The specificity and selectivity of the method relied on the monitoring of one quantitative ion and two qualitative ions at the specific retention times of each analyte and internal standard. ACO-*d*_6_ was employed as the IS for ACE, ACO, DA, ACRL, FUR, and PhCHO, while HEX-*d*_12_ was used for FCHO, 2=C4AL, C5AL, GO, and MDA. Standard addition calibration with IS curves covered a concentration range from LOQ to 3 μg/mL, yielding excellent determination coefficients (≥0.9991). Method precision was evaluated through quintuplicate intra- and interday assays at three concentration levels using QCs, with RSDs of ≤1.59% and 7.55%, respectively. Accuracy was assessed at the same levels by quintuplicate analyses, achieving recoveries between 90.0 and 107.1%. Instrumental limits and matrix effect data are summarized in Table 3. Although matrix effects were observed by some analytes, no matrix interferences were detected in the GC-MS chromatograms (Figure 7). This confirmed the suitability of the standard addition calibration approach for accurately estimating the occurrence of these analytes in real samples.

**Figure 7:**
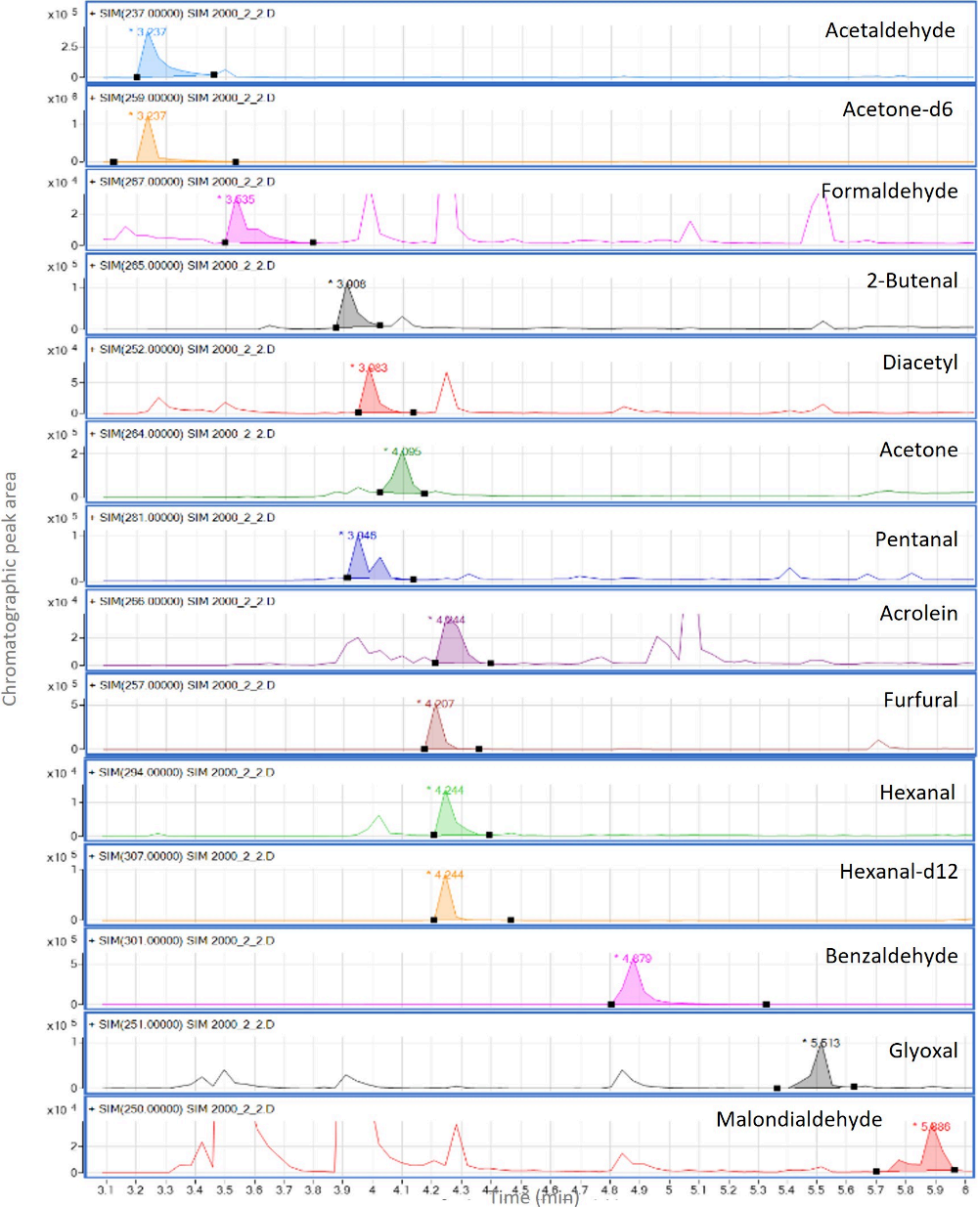
Chromatogram of a nonroasted almond-based beverage spiked at 2 μg/mL.

**Table 3:** Instrumental Limits and Matrix Effect

	Standard Addition Calibration		External Calibration				
Analyte	m	b	m	b	Matrix effect^a^ (%)	LOD^b^ (ng/mL)	LOQ^b^ (ng/mL)
Acetaldehyde	0.0007	0.4806	0.0064	0.2305	11%	0.5	2
Formaldehyde	0.0241	9.8611	0.0028	–0.3528	861%	0.2	1
2-Butenal	0.0198	0.2208	0.0027	–0.4846	733%	1	3
Diacetyl	0.015	1.1151	0.0142	0.5237	106%	1.5	5
Acetone	0.00005	0.0001	0.0006	0.1106	8%	1	3
Pentanal	0.0002	0.1655	0.001	0.0592	20%	1	3
Acrolein	0.00001	0.0004	0.00001	0.0182	100%	5	15
Furfural	0.0002	0.0173	0.0008	–0.0173	25%	1.2	4
Hexanal	0.0015	0.0002	0.0005	0.0782	300%	0.5	2
Benzaldehyde	0.0002	0.0017	0.0003	0.0084	67%	1.1	4
Glyoxal	0.0176	0.2913	0.01	–0.0797	176%	3	10
Malondialdehyde	0.0043	1.0496	0.0041	0.0125	105%	6	20

a Estimated by comparison of the slope of both calibration curves for each analyte.

b Instrumental limit estimated with pure standard and in the absence of sample. Linear dynamic range: 200–3000 ng/mL.

Table S2 compares previously published DLLME procedures for determining carbonyls in food matrixes.^24,26,32,40–44^ In comparison with previously published methods, our approach enables the simultaneous extraction and derivatization of 14 carbonyls from diverse chemical families within 20 min, whereas other methodologies achieve faster extractions (0.7–5 min) but are restricted to single compounds or, at most, three analytes belonging to the same family.^26,32,40,43,44^ In terms of analytical performance, the method developed here achieves comparable or lower determination limits than those reported for most multianalyte GC-MS approaches.^24,32,41^ Although previous studies reported lower determination limits than ours, like Zhang et al. in their HPLC–UV analysis of FCHO, ACE, C5AL, HEX, and other carbonyls in drinking water and alcoholic beverages^42^ and Xu et al. in their HPLC-UV determination of FCHO in beverages^26^ and in their HPLC-FD determination of GO in alcoholic beverages after derivatization with 3,4-diaminopyridine,^40^ these results likely reflect the lower matrix complexity of those samples compared with PBBs. In contrast, our approach offers clear advantages by delivering reliable sensitivity in a much more complex food matrix while covering a considerably broader set of carbonyl targets within a single run.

Precision values reported in earlier studies are broadly comparable to those obtained here.^24,26,32,40–44^ However, the method proposed in this work demonstrates superior accuracy with recoveries consistently closer to 100%. This improvement can be ascribed to the use of derivatization coupled with MS detection, which strengthens selectivity and sensitivity, reduces false positives, and ensures more reliable quantification, as shown in recent MS-based carbonyl determinations.^45,46^

### Sustainability and Viability Evaluation of the US-DLLME Procedure

The AGREEprep assessment, performed with the open-access AGREEprep software, was used to evaluate the environmental impact of our sample-preparation methods. This tool identifies strengths and weaknesses across green chemistry criteria and supports the design of more eco-friendly workflows.^29,30^ In parallel, the practical applicability of the analytical method was evaluated using the BAGI online app,^31^ which considers ten attributes grouped into two categories: analytical determination (e.g., type of analysis, number of analytes, required instrumentation) and sample preparation (e.g., sample-handling capacity, reagents used, degree of automation). Figure 8 presents the AGREEprep results for the environmental assessment (A) and the BAGI results (B) for the DLLME procedure.

**Figure 8:**
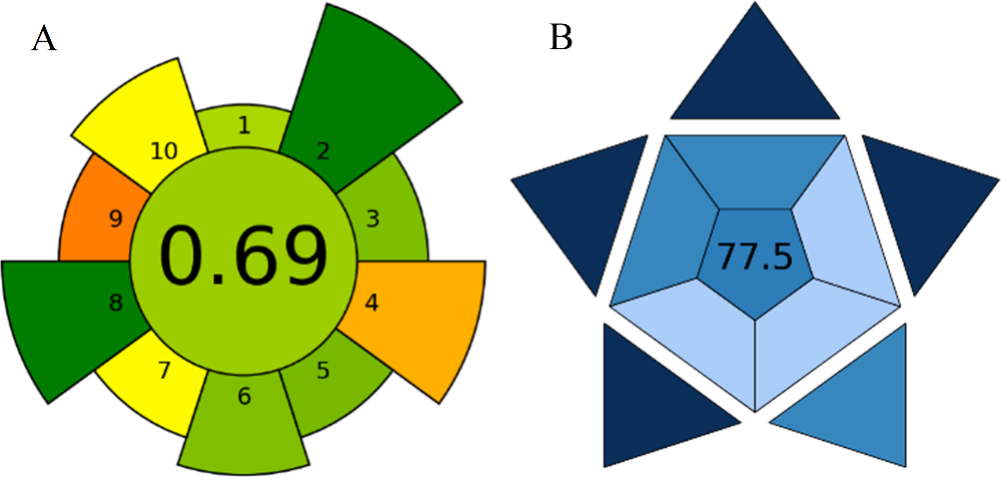
Environmental impact assessment using the AGREEprep software (A) and practical applicability using the BAGI online tool (B) of the US-DLLME method. Scoring criteria are described in the text.

AGREEprep evaluates ten green analytical chemistry criteria: (1) in situ sample preparation, (2) use of safer solvents and reagents, (3) preference for sustainable, reusable, and renewable materials, (4) waste minimization, (5) reduced consumption of sample, chemicals, and materials, (6) maximized sample throughput, (7) integration of steps and promotion of automation, (8) minimized energy consumption, (9) selection of the greenest possible postsample-preparation configuration for analysis, and (10) operator safety. Scores range from 0 (least sustainable) to 1 (most sustainable), with higher values indicating a better environmental performance. The BAGI online tool considers ten attributes: (1) type of analysis, (2) number of analytes determined simultaneously, (3) analytical technique and instrumentation required, (4) number of samples that can be treated simultaneously, (5) sample-preparation process, (6) sample throughput per hour, (7) reagents and materials used, (8) need for preconcentration, (9) degree of automation, and (10) sample amount. Attributes are color-coded from white (low applicability, score 2.5) to dark blue (high applicability, score 10).

The US-DLLME-GC-MS method achieved an overall AGREEprep score of 0.69. Strong performance was observed for the absence of hazardous materials (score 1.0), the single-step design, and low energy consumption (score 1.0). Additional favorable factors were the economy of sample size (0.5 g; score 0.77), high sample throughput (up to 24 simultaneous experiments per hour; score 0.75), and the use of sustainable/renewable reagents and materials (>75%; score 0.75). Limitations were associated with the waste generated (5.79 mL per sample; score 0.35) and the use of mass spectrometry as the post-sample-preparation configuration (score 0.25).

Similarly, BAGI evaluates ten key attributes grouped into analytical determination and sample preparation (as listed above). Each attribute is color-coded on a 0–100 scale, where dark blue indicates high applicability (score 10) and white indicates low applicability (score 2.5). The US-DLLME-GC-MS method obtained an overall BAGI score of 77.5. High-scoring elements included simultaneous confirmatory and quantitative analysis (factor 1, score 10), multielement/multianalyte capability (factor 2, score 10), simultaneous sample preparation (factor 4, score 10), and miniaturized extraction (factor 5, score 10).

The comparison of methodologies in Table S3 highlights current efforts to enhance the DLLME sustainability. A principal challenge is the need for derivatization to improve detectability for most carbonyls.^11,45^ Notably, FUR and HMF have been determined by HPLC-UV without derivatization using manual agitation; however, multianalyte approaches typically required derivatization at different temperatures in microwave-, ultrasonic-, or vortex-assisted workflows, and the technique itself is generally less selective than GC–MS. Solvent selection is another critical level. ACN is a common dispersant but is problematic from a toxicity standpoint; MeOH is also less favorable, in terms of sustainability. EtOH and IPA represent more sustainable alternatives: EtOH, especially when produced as bioethanol, has a lower carbon footprint and is less toxic, with lower vapor pressure, thus reducing laboratory exposure and disposal concerns.^47^ IPA is similarly regarded as a greener alternative to MeOH and ACE, with a lower environmental risk ranking and bio-based production routes available and less expensive than EtOH.^36^ The extractant solvent remains the hardest to replace.^11^ Chloroform and dichloromethane are still widely used due to efficiency, despite their high toxicity.^11^ Explored alternatives include ionic liquids (e.g., trihexyltetradecylphosphonium chloride),^26,43^ which offer lower volatility and improved safety profiles but pose practical challenges, especially high viscosity and handling difficulties.^48^ Regarding hydrocarbon/alcohol-based extractants, greener low-density solvents such as isooctane and 1-octanol have been tested for carbonyl extraction with success.^11,41,44^ IBA, identified in solvent selection guides as a greener ester with low toxicity, biodegradability, and potential for bio-based production,^48^ was selected here as a more sustainable extractant option. This choice balances improved environmental compatibility with the suitable extraction performance that we observed in our settings.

### Carbonyl Compounds Occurrence in Plant-Based Beverages

Occurrence results for the 12 target carbonyl compounds are summarized in Table S3. ACE was consistently detected in most matrixes (detection frequency 90–100%), with positive-only medians ranging from ∼790 ng/mL in minor crops to ∼1060 ng/mL in almond and coconut. FCHO, in contrast, was detected infrequently (≤30% of samples) and showed variable positive medians, from 518.8 ng/mL in mixed formulations to >1500 ng/mL in oat drinks and >1021 ng/mL in almond drinks. Several other carbonyls, including 2=C4Al, ACRL, and MDA, showed detection frequencies ≤40% in most groups and positive-only medians between ∼300 and 1600 ng/mL; their sporadic detection suggests a limited role in discriminating matrixes. Four analytes displayed clear matrix-dependent fingerprints. DA was detected in nearly all soy, almond, oat, and mixed samples (≥93–100% detection), with positive medians of ∼2915 ng/mL in soy, ∼1142 ng/mL in almond, ∼973 ng/mL in oat, and ∼1468 ng/mL in mixed formulations; coconut showed a lower median (784 ng/mL) with only 50% detection. FUR was detected across all groups and reached its highest positive median in coconut (∼2512 ng/mL), followed by soy (∼2016 ng/mL), rice (∼1824 ng/mL), oat (∼1556 ng/mL), mixed (∼1649 ng/mL), and almond (∼1254 ng/mL). HEX, a marker of lipid oxidation, was detected in almost all matrixes except mixed formulation, with positive medians around 350 ng/mL in almond, oat, and coconut and higher values in minor crops (∼593 ng/mL). GO distinguished almond and mixed formulations (positive medians ∼345 and ∼309 ng/mL; detection frequencies 80% and 43%, respectively), whereas soy, oat, coconut, rice, and minor crops showed either very low or no detections.

When compared with published data, the magnitude and distribution of some analytes were consistent while others diverged. Pucci et al. reported GO levels between 47 and 439 μg/100 mL across PBBs, in line with the values reported here, confirming GO as a robust discriminant for almond- and rice-based beverages.^9^ By contrast, FUR was detected in only almond drinks (∼1 μg/100 mL) in that study, whereas our method quantified it in all matrixes at concentrations between 1254 and 2512 ng/mL, particularly enriched in coconut. Similarly, DA was quantified by Pucci et al. in soy and oat drinks at 9–73 μg/100 mL but at substantially lower levels than in our data set (973–2915 ng/mL).^9^ In contrast, our positive-only medians for HEX (296–593 ng/mL) aligned well with the ranges reported by Manousi and Zachariadis for nut-based beverages (254–4494 ng/mL), supporting its role as a lipid oxidation marker.^49^ Multivariate analysis of the log-relative profiles confirmed these tendencies (Figure 9).

**Figure 9:**
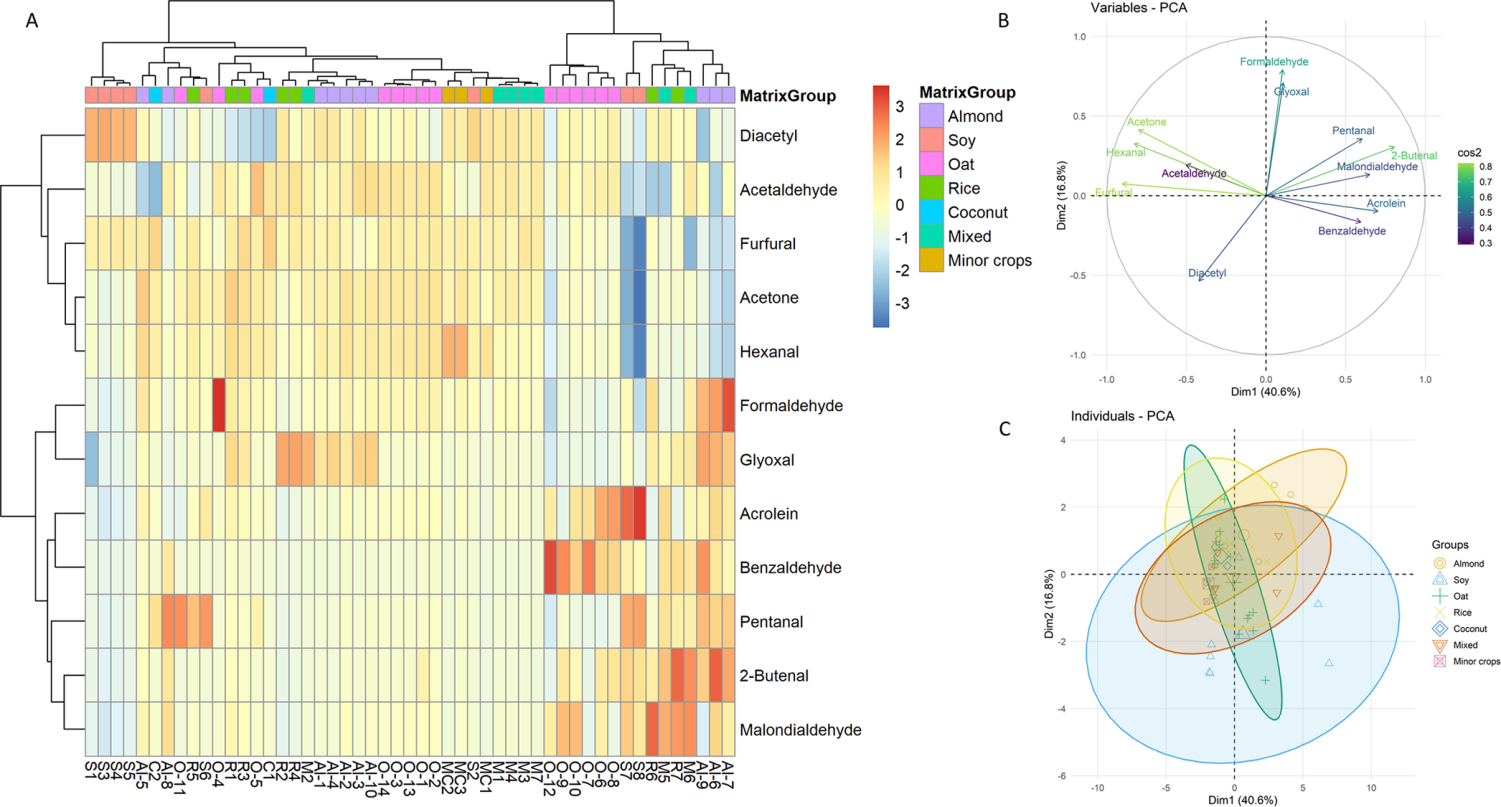
Multivariate analysis of carbonyl profiles in plant-based beverages: (A) hierarchical clustering heatmap, (B) PCA loading plot, and (C) PCA score plot with 95% ellipses.

PCA accounted for 57% of the total variance in the first two dimensions and revealed clear tendencies: soy samples clustered toward diacetyl, coconut toward furfural, oat toward hexanal, and almond and rice toward glyoxal, while mixed formulations and minor crops showed no reproducible separation. The loading plot corroborated the discriminant role of these four carbonyls, in contrast with background aldehydes such as acrolein, 2-butenal, malondialdehyde, and formaldehyde, which contributed little to overall variance. The hierarchical clustering heatmap further supported this structure by grouping analytes into two main clusters: (i) discriminant markers including diacetyl, furfural, hexanal, and acetaldehyde and (ii) glyoxal/formaldehyde, while noninformative aldehydes were relegated to a secondary cluster. Importantly, nonparametric testing reinforced these patterns: Kruskal–Wallis and Dunn’s post hoc comparisons confirmed significant differences for diacetyl, furfural, hexanal, and glyoxal across matrixes (*p* < 0.05), and effect size analysis indicated moderate-to-strong discriminatory power. Finally, PERMANOVA demonstrated that the overall carbonyl fingerprints differed significantly between matrixes (*R* ^2^ = 0.21, *p* = 0.009), confirming that, despite partial overlap, the carbonyl profile of plant-based beverages contains sufficient information for matrix classification.

Assessing the presence of reactive carbonyl species in plant-based beverages is a valuable approach for estimating their potential toxic impact on human health and well-being. Although many food contaminants have been extensively studied and maximum detection limits are established in European legislation, most carbonyl compounds are not covered by Regulation (EU) 2023/915 on maximum levels for certain contaminants in food.^50^

Some carbonyl contaminants arise in foods due to migration from packaging materials. In these cases, the specific migration limits (SMLs) are regulated under Regulation (EU) 10/2011 (specific for formaldehyde-15 mg/kg food and acetaldehyde-6 mg/kg food).^51^ Given this context, it is important to monitor the presence of these compounds in food products and relate their concentrations to the available toxicological data. Accordingly, Table S4 summarizes the estimated daily intake of reactive carbonyl species in the average adult in Europe (assumed body weight: 75 kg) after consuming a 250 mL serving of the plant-based beverages studied. The levels detected appear to be safe, as they are significantly below the established TDI and ADI values (Table 1) and remain under *No Observed Adverse Effect Level* (NOAEL) thresholds.^13–19^

## Conclusions

This work established an optimized US-DLLME–derivatization procedure for the simultaneous extraction of carbonyls from PBBs with a reduced environmental impact. Among the derivatization reagents tested, PFBHA was selected as the most suitable, providing consistently higher chromatographic responses and a better peak resolution. Acidification at pH 2 using a reduced amount of HCl was confirmed as the most effective condition, minimizing buffer interferences and ensuring stable oxime formation. Reaction temperature was shown to exert analyte-dependent effects, with 40 °C offering overall improved performance and stability, and was therefore adopted in the optimized protocol.

Evaluation of extraction solvents identified IBA as the best compromise between extraction efficiency and sustainability. Although DMC also provided strong signals, IBA consistently delivered superior responses across the analyte set and offered practical advantages when used as both an extractant and a defatting solvent. For dispersants, IPA outperformed ACN by yielding higher and more reproducible responses while also representing a greener option. Optimization of solvent volumes by CDD confirmed a robust operating window, with 90 μL of IBA and 1.0 mL of IPA selected as optimal conditions.

The optimized method demonstrated excellent analytical performance, with low limits of determination, high linearity, precision, and accuracy and recoveries consistently close to 100%. Comparison with previously reported DLLME procedures shows that this approach achieves similar or superior performance while incorporating greener solvent choices and reduced hazardous reagents. AGREEprep (score 0.69/1) and BAGI (score 77.5/100) assessments further highlighted the environmental and practical benefits of the method, with strong scores for low toxicity, energy efficiency, and sample throughput.

Occurrence analysis revealed that, although most carbonyls such as formaldehyde, 2-butanal, acrolein, and malondialdehyde were consistently detected at low or near-LOD levels, four analytes provided clear matrix-dependent fingerprints. Diacetyl was enriched in soy, furfural in coconut, hexanal in oat, and glyoxal in almond and rice. These discriminant patterns, supported by multivariate clustering, demonstrate that carbonyl profiles offer a useful chemical basis to differentiate plant-based beverages despite partial overlap among matrixes.

Overall, the developed US-DLLME-GC-MS method provides a selective, accurate, and reproducible strategy for carbonyl determination in PBBs, while advancing the principles of green analytical chemistry.

## Supplementary Material



## Notes

Figures showing extracted ion chromatograms of target carbonyls derivatized with *O*-(*tert*-butyldimethylsilyl) hydroxylamine, *O*-(2,3,4,5,6-pentafluorobenzyl) hydroxylamine hydrochloride, methoxylamine, and hydroxylamine; protein precipitation by acetonitrile, isopropanol, methanol, and ethanol; delta weight plots from asymmetrical screening design; total effects from asymmetrical screening design; and surface responses from central composite design and tables showing the precursor ion of the carbonyls derivatized with *O*-(2,3,4,5,6-pentafluorobenzyl) hydroxylamine hydrochloride (PFBHA), *O*-(*tert*-butyldimethylsilyl) hydroxylamine (TBS-ONH_2_), hydroxylamine (NH_2_OH), and methoxylamine hydrochloride (MOX); a comparison of published dispersive liquid–liquid microextraction procedures for carbonyl compounds determination in food; and carbonyl occurrence in plant-based beverages (PDF)

## References

[1] McClements D. J., Newman E., McClements I. F. (2019). Plant-Based
Milks: A Review of the
Science Underpinning Their Design, Fabrication, and Performance. Compr. Rev. Food Sci. Food Saf..

[2] Xiao L., Sun Y., Tsao R. (2022). Paradigm Shift
in Phytochemicals Research: Evolution
from Antioxidant Capacity to Anti-Inflammatory Effect and to Roles
in Gut Health and Metabolic Syndrome. J. Agric.
Food Chem..

[3] de
Oliveira Ribeiro A. P., dos Santos Gomes F., dos Santos K. M. O., da Matta V. M., de Araujo Santiago M.
C. P., Conte C., Walter E. H. M. (2020). Development of a Probiotic Non-Fermented Blend Beverage
with Juçara Fruit: Effect of the Matrix on Probiotic Viability
and Survival to the Gastrointestinal Tract. LWT.

[4] Pérez-Rodríguez M. L., Serrano-Carretero A., García-Herrera P., Cámara-Hurtado M., Sánchez-Mata M. C. (2023). Plant-based beverages as milk alternatives?
Nutritional and functional approach through food labelling. Food Research International.

[5] Ministerio de Agricultura, Pesca y Alimentación . Informe del Consumo Alimentario en España 2023; Gobierno de España: Madrid, 2024. https://www.mapa.gob.es/es/alimentacion/temas/consumo-tendencias/informe_2023_alta_tcm30-685877.pdf (accessed August 27, 2025).

[6] European Union . Regulation (EU) 2018/848 of the European Parliament and of the Council of 30 May 2018 on Organic Production and Labelling of Organic Products and Repealing Council Regulation (EC) No 834/2007. Off. J. Eur. Union 2018, L150, 1–92. http://data.europa.eu/eli/reg/2018/848/oj (accessed August 27, 2025).

[7] Sharma N., Yeasmen N., Dube L., Orsat V. (2024). A Review on Current
Scenario and Key Challenges of Plant-Based Functional Beverages. Food Biosci..

[8] Adimas Z. T., Abera B. D. (2025). Effect of Roasting Time and Temperature on the Physicochemical
and Sensory Properties of Plant Beverage from Groundnut. Appl. Food Res..

[9] Pucci M., Akıllıoğlu H. G., Bevilacqua M., Abate G., Lund M. N. (2024). Investigation of
Maillard Reaction
Products in Plant-Based Milk Alternatives. Food
Res. Int..

[10] Moretto L., Tonolo F., Folda A., Scalcon V., Bindoli A., Bellamio M., Feller E., Rigobello M. P. (2021). Comparative
Analysis of the Antioxidant Capacity and Lipid and Protein Oxidation
of Soy and Oats Beverages. Food Prod. Process.
Nutr..

[11] Custodio-Mendoza J. A., Ares-Fuentes A. M., Carro A. M. (2023). Innovative Solutions for Food Analysis:
Microextraction Techniques in Lipid Peroxidation Product Detection. Separations.

[12] World Health Organization . IARC Monographs on the Identification of Carcinogenic Hazards to Humans. 2023. https://monographs.iarc.who.int/list-of-classifications/ (accessed August 27, 2025).

[13] European
Food Safety Authority (EFSA) (2007). Opinion of the Scientific Panel on Food Additives, Flavourings, Processing
Aids and Materials in Contact with Food (AFC) Related to Use of Formaldehyde
as a Preservative during the Manufacture and Preparation of Food Additives. EFSA J..

[14] Center for Drug Evaluation and Research . M7­(R2) Addendum: Application of the Principles of the ICH M7 Guideline; U.S. Food and Drug Administration: Silver Spring, MD, 2022. https://www.fda.gov/media/157451/download (accessed August 27, 2025).

[15] Van Andel, I. ; Sleijffers, A. ; Schenk, E. ; Rambali, B. ; Wolterink, G. ; Vleeming, W. ; van Amsterdam, J. G. C. Adverse Health Effects of Cigarette Smoke: Aldehydes Crotonaldehyde, Butyraldehyde, Hexanal and Malonaldehyde; Dutch National Institute of Public Health and the Environment: Utrecht, The Netherlands, 2006. http://hdl.handle.net/10029/7336 (accessed August 27, 2025).

[16] Clark S., Winter C. K. (2015). Diacetyl in Foods: A Review of Safety and Sensory Characteristics. Compr. Rev. Food Sci. Food Saf..

[17] Papastergiadis A., Fatouh A., Jacxsens L., Lachat C., Shrestha K., Daelman J., Kolsteren P., Van Langenhove H., De Meulenaer B. (2014). Exposure Assessment of Malondialdehyde,
4-Hydroxy-2-(E)-Nonenal
and 4-Hydroxy-2-(E)-Hexenal through Specific Foods Available in Belgium. Food Chem. Toxicol..

[18] Gomes, R. ; Meek, M. K. Acrolein. Concise International Chemical Assessment Document 43; World Health Organization: Geneva, 2002. https://iris.who.int/server/api/core/bitstreams/fd793162-06d5-45c7-acc8-a9c9fe53439d/content (accessed August 27, 2025).

[19] Younes M., Aquilina G., Castle L., Engel K. H., Fowler P., Mennes W., EFSA
Panel on Food Additives and Flavourings (FAF) (2021). Scientific Opinion
on Flavouring Group Evaluation 13 Revision 3 (FGE.13Rev3): Furfuryl
and Furan Derivatives with and without Additional Side-Chain Substituents
and Heteroatoms from Chemical Group 14. EFSA
J..

[20] Ghani M. A., Barril C., Bedgood D. R., Prenzler P. D. (2017). Measurement
of Antioxidant Activity with the Thiobarbituric Acid Reactive Substances
Assay. Food Chem..

[21] Armenta S., Garrigues S., Esteve-Turrillas F. A., de la Guardia M. (2019). Green Extraction
Techniques in Green Analytical Chemistry. TrAC,
Trends Anal. Chem..

[22] López-Lorente Á. I., Pena-Pereira F., Pedersen-Bjergaard S., Zuin V. G., Ozkan S. A., Psillakis E. (2022). The Ten Principles
of Green Sample Preparation. TrAC, Trends Anal.
Chem..

[23] Gonçalves L. M., Magalhães P. J., Valente I. M., Pacheco J. G., Dostálek P., Sýkora D., Rodrigues J. A., Barros A. A. (2010). Analysis of Aldehydes
in Beer by Gas-Diffusion Microextraction: Characterization by High-Performance
Liquid Chromatography-Diode-Array Detection-Atmospheric Pressure Chemical
Ionization-Mass Spectrometry. J. Chromatogr.
A.

[24] Custodio-Mendoza J. A., Aja-Macaya J., Valente I. M., Rodrigues J. A., Almeida P. J., Lorenzo R. A., Carro A. M. (2020). Determination of
Malondialdehyde, Acrolein and Four Other Products of Lipid Peroxidation
in Edible Oils by Gas-Diffusion Microextraction Combined with Dispersive
Liquid–Liquid Microextraction. J. Chromatogr.
A.

[25] Moreira N., Araújo A. M., Rogerson F., Vasconcelos I., De Freitas V., de Pinho P. G. (2019). Development and Optimization of a
HS-SPME-GC-MS Methodology to Quantify Volatile Carbonyl Compounds
in Port Wines. Food Chem..

[26] Xu X., Su R., Zhao X., Liu Z., Li D., Li X., Wang Z. (2011). Determination of Formaldehyde
in Beverages Using Microwave-Assisted
Derivatization and Ionic Liquid-Based Dispersive Liquid–Liquid
Microextraction Followed by High-Performance Liquid Chromatography. Talanta.

[27] U.S. Food and Drug Administration . Bioanalytical Method Validation Guidance for Industry; U.S. Department of Health and Human Services: Silver Spring, MD, 2018; pp 1–41. https://www.fda.gov/files/drugs/published/Bioanalytical-Method-Validation-Guidance-for-Industry.pdf (accessed August 25, 2025).

[28] U.S. Food and Drug Administration . Guidelines for the Validation of Chemical Methods in Food, Feed, Cosmetics, and Veterinary Products; Food and Drug Administration: Silver Spring, MD, 2019; pp 1–39. https://www.fda.gov/media/81810/download (accessed August 25, 2025).

[29] Pena-Pereira F., Tobiszewski M., Wojnowski W., Psillakis E. (2022). A Tutorial
on AGREEprep: An Analytical Greenness Metric for Sample Preparation. Adv. Sample Prep..

[30] Wojnowski W., Tobiszewski M., Pena-Pereira F., Psillakis E. (2022). AGREEprep
– Analytical Greenness Metric for Sample Preparation. TrAC, Trends Anal. Chem..

[31] Manousi N., Wojnowski W., Płotka-Wasylka J., Samanidou V. (2023). Blue Applicability
Grade Index (BAGI) and Software: A New Tool for the Evaluation of
Method Practicality. Green Chem..

[32] Custodio-Mendoza J. A., Caamaño-Fernandez C., Lage M. A., Almeida P. J., Lorenzo R. A., Carro A. M. (2022). GC–MS
Determination of Malondialdehyde,
Acrolein, and 4-Hydroxy-2-Nonenal by Ultrasound-Assisted Dispersive
Liquid–Liquid Microextraction in Beverages. Food Chem..

[33] Wang S., Nawale G. N., Kadekar S., Oommen O. P., Jena N. K., Chakraborty S., Varghese O. P. (2018). Saline Accelerates Oxime Reaction
with Aldehyde and Keto Substrates at Physiological pH. Sci. Rep..

[34] Larsen D., Kietrys A. M., Clark S. A., Park H. S., Ekebergh A., Kool E. T. (2018). Exceptionally Rapid
Oxime and Hydrazone Formation Promoted
by Catalytic Amine Buffers with Low Toxicity. Chem. Sci..

[35] Gałuszka A., Migaszewski Z., Konieczka P., Namieśnik J. (2012). Analytical
Eco-Scale for Assessing the Greenness of Analytical Procedures. TrAC, Trends Anal. Chem..

[36] Tobiszewski M., Namieśnik J., Pena-Pereira F. (2017). Environmental
Risk-Based Ranking
of Solvents Using the Combination of a Multimedia Model and Multi-Criteria
Decision Analysis. Green Chem..

[37] Pacheco-Fernandez I., Pino V. (2019). Green Solvents in Analytical Chemistry. Curr.
Opin. Green Sustain. Chem..

[38] Yang Y., Cruickshank C., Armstrong M., Mahaffey S., Reisdorph R., Reisdorph N. (2013). New Sample
Preparation Approach for Mass Spectrometry-Based
Profiling of Plasma Results in Improved Coverage of Metabolome. J. Chromatogr. A.

[39] Polson C., Sarkar P., Incledon B., Raguvaran V., Grant R. (2003). Optimization of Protein Precipitation Based upon Effectiveness of
Protein Removal and Ionization Effect in Liquid Chromatography–Tandem
Mass Spectrometry. J. Chromatogr. B.

[40] Rodríguez-Cáceres M. I., Palomino-Vasco M., Mora-Diez N., Acedo-Valenzuela M. I. (2017). Dispersive
Liquid–Liquid Microextraction for a Rapid Determination of
Glyoxal in Alcoholic Beverages. Talanta.

[41] Silva A. R., Custodio-Mendoza J. A., Santos J. R., Almeida P. J., Rodrigues J. A., Carro A. M. (2025). Green Solvents in Dispersive Liquid–Liquid Microextraction
for the Determination of Carbonyl Compounds in Coffee Extracts. J. Chromatogr. A.

[42] Zhang K., Guo R., Wang Y., Wang J., Nie Q., Zhu G. (2024). Terpenes-Based
Hydrophobic Deep Eutectic Solvents for Dispersive Liquid–Liquid
Microextraction of Aliphatic Aldehydes in Drinking Water and Alcoholic
Beverages. Chemosphere.

[43] Nascimento C. F., Brasil M. A., Costa S. P., Pinto P. C., Saraiva M. L. M., Rocha F. R. (2015). Exploitation of
Pulsed Flows for On-Line Dispersive
Liquid–Liquid Microextraction: Spectrophotometric Determination
of Formaldehyde in Milk. Talanta.

[44] Madani-Tonekaboni M., Kamankesh M., Mohammadi A. (2015). Determination of Furfural and Hydroxymethyl
Furfural from Baby Formula Using Dispersive Liquid–Liquid Microextraction
Coupled with High-Performance Liquid Chromatography and Method Optimization
by Response Surface Methodology. J. Food Compos.
Anal..

[45] Dator R., Carrà A., Maertens L., Guidolin V., Villalta P. W., Balbo S. (2017). A High Resolution/Accurate Mass (HRAM) Data-Dependent MS^3^ Neutral Loss Screening, Classification, and Relative Quantitation
Methodology for Carbonyl Compounds in Saliva. J. Am. Soc. Mass Spectrom..

[46] Thanayutsiri T., Mantadilok S., Sapsin J., Tungwattanaviboon T., Wongwatanasin J., Opanasopit P., Ngawhirunpat T., Rojanarata T. (2025). Development
of a Green and Rapid Ethanol-Based HPLC
Assay for Aspirin Tablets and Feasibility Evaluation of Domestically
Produced Bioethanol in Thailand as a Sustainable Mobile Phase. Green Process. Synth..

[47] Nie L., Cai C., Guo R., Yao S., Zhu Z., Hong Y., Guo D. (2022). Ionic Liquid-Assisted DLLME and SPME for the Determination of Contaminants
in Food Samples. Separations.

[48] Sheldon R. A. (2019). The Greening
of Solvents: Towards Sustainable Organic Synthesis. Curr. Opin. Green Sustain. Chem..

[49] Manousi N., Zachariadis G. A. (2019). Determination
of Volatile Compounds in Nut-Based Milk
Alternative Beverages by HS-SPME Prior to GC-MS Analysis. Molecules.

[50] European Commission . (2023, April 25). Commission Regulation (EU) 2023/915 on maximum levels for certain contaminants in food (OJ L 119). EUR-Lex. https://eur-lex.europa.eu/eli/reg/2023/915/oj/eng.

[51] European Commission . (2011, January 14). Commission Regulation (EU) No. 10/2011 on plastic materials and articles intended to come into contact with food (OJ L 12, 15 Jan 2011). EUR-Lex. https://eur-lex.europa.eu/legal-content/EN/ALL/?uri=celex%3A32011R0010.

