# Supplementary material for: Green Ultrasound-Assisted Dispersive Liquid–Liquid Microextraction Coupled to GC–MS for Simultaneous Determination of Lipid-Peroxidation- and Maillard-Derived Carbonyl Compounds in Plant-Based Beverages
Source: ACS Meas Sci Au. 2025 Nov 3;5(6):1010–22. doi: 10.1021/acsmeasuresciau.5c00133 (PMC12715736; doi:10.1021/acsmeasuresciau.5c00133)
Supplement: Supplementary file 1 [file tg5c00133_si_001.pdf]

Supplementary Materials

Green Ultrasound-Assisted Dispersive Liquid–Liquid Microextraction Coupled to GC–MS for Simultaneous Determination of Lipid Peroxidation- and Maillard-Derived Carbonyl Compounds in Plant-Based Beverages

Jorge A. Custodio-Mendoza [a*], Antía Villanueva [b], Agata Antoniewska-Krzeska [c], Rosa Pérez-Gregorio [a], Elena Martínez-Carballo [a], María Llompart [d], and Antonia María Carro-Díaz [b,e,f]

[a] *Instituto de Agroecoloxía e Alimentación (IAA) – Food and Health Omics, Universidade de Vigo, Campus Auga, 32004 Ourense, Spain*

[b] *Department of Analytical Chemistry, Nutrition and Food Science, Faculty of Chemistry, Universidade de Santiago de Compostela (USC), 15782, Santiago de Compostela, Spain*

[c] *Institute of Human Nutrition Sciences, Faculty of Human Nutrition, Warsaw University of Life Sciences, Nowoursynowska 159c, 02-776 Warsaw, Poland*

[d] *CRETUS – Department of Analytical Chemistry, Nutrition and Food, USC, 15782 Santiago de Compostela, Spain*

[e] *Health Research Institute of Santiago de Compostela (IDIS), USC, 15782 Santiago de Compostela, Spain*

f Instituto de Materiais (iMATUS), USC, 15782 Santiago de Compostela, Spain

*Email: jorgeantonio.custodio@uvigo.gal

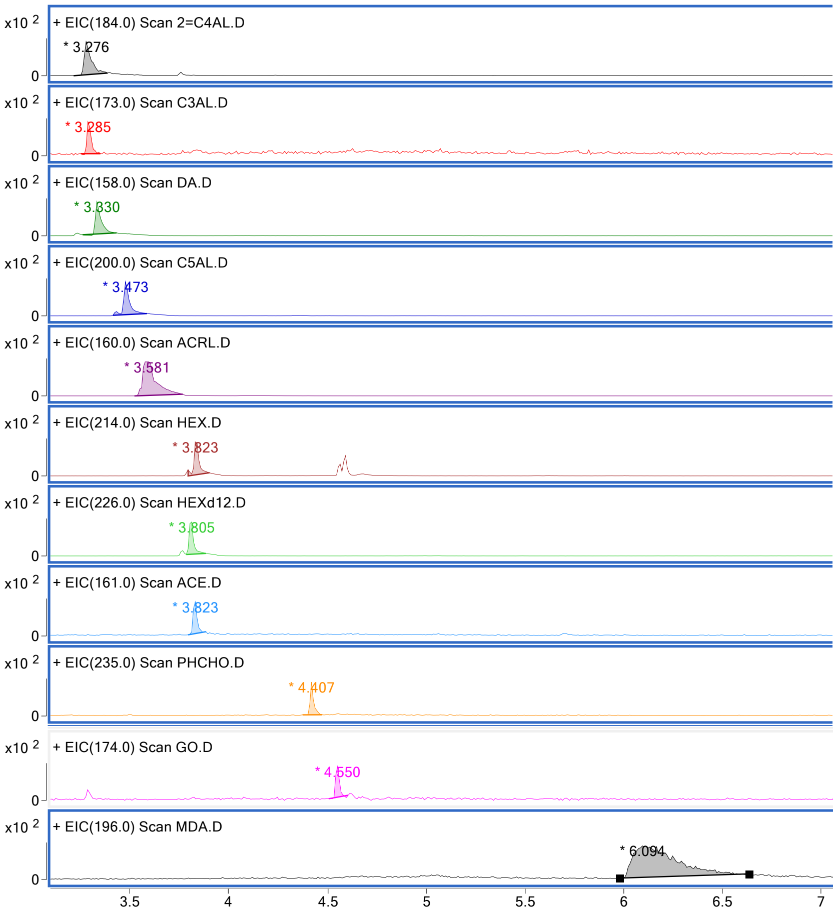

Figure S1. Extracted ion chromatogram of target carbonyls derivatized with O-(tert-butyldimethylsilyl) hydroxylamine. 2=C4AL, 2-butenal; C3Al, propanal; DA, diacetyl; C5AL, pentanal; ACRL, acrolein; HEX, hexanal; HEXd12, hexanal deuterated; ACE, acetaldehyde; PhCHO, benzaldehyde; GO, glyoxal; MDA, malondialdehyde.

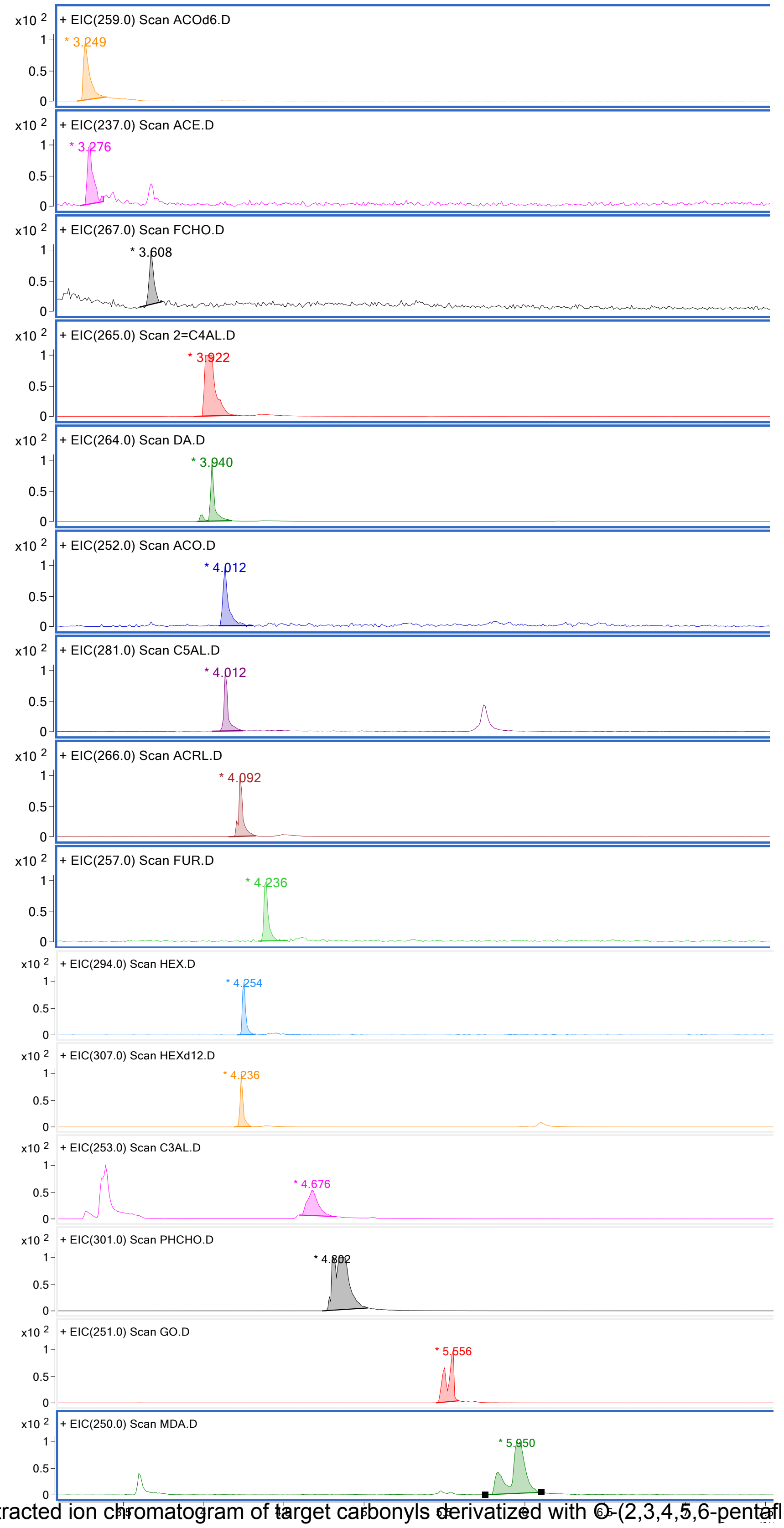

Figure S2. Extracted ion chromatogram of target carbonyls derivatized with O-(2,3,4,5,6-pentafluorobenzyl) hydroxylamine hydrochloride. ACOd6, acetone deuterated; ACE, acetaldehyde; FCHO, formaldehyde; 2=C4AL, 2-butenal; DA, diacetyl; ACO, acetone; C5AL, pentanal; ACRL, acrolein; FUR, furfural; HEX, hexanal; HEXd12, hexanal deuterated; C3AL, propanal; PhCHO, benzaldehyde; GO, glyoxal; MDA, malondialdehyde.

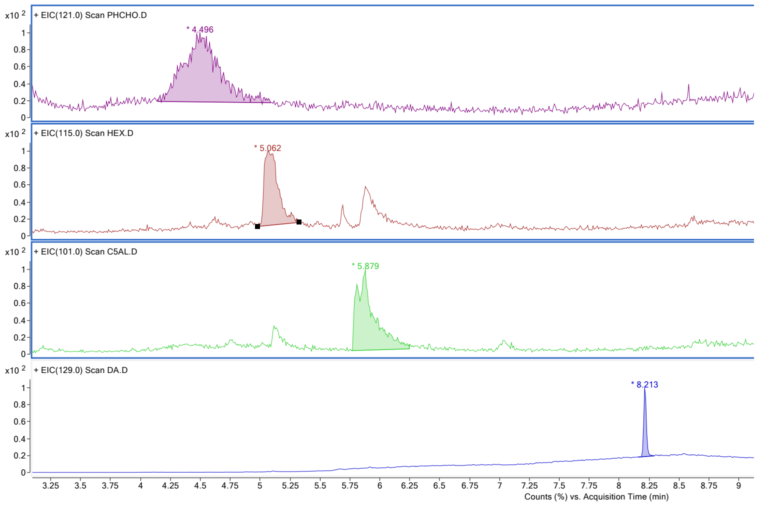

Figure S3. Extracted ion chromatogram of target carbonyls derivatized with methoxylamine. PhCHO, benzaldehyde; C5AL, pentanal; HEX, hexanal; DA, diacetyl.

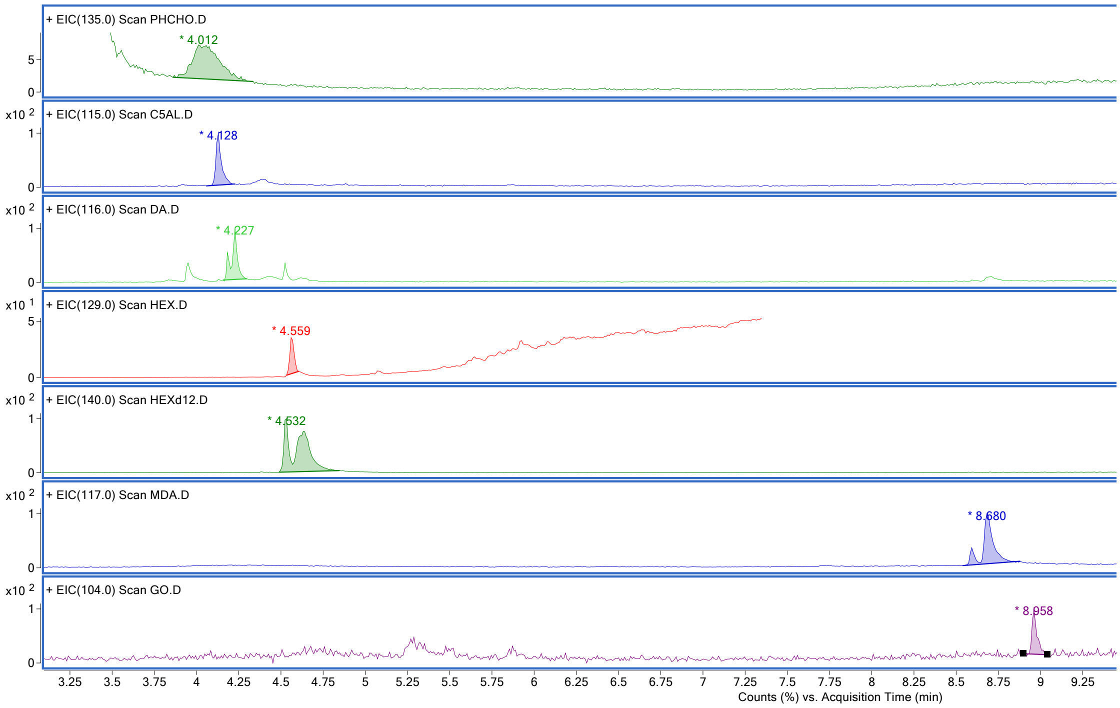

Figure S4. Extracted ion chromatogram of target carbonyls derivatized with hydroxylamine. PhCHO, benzaldehyde; C5AL, pentanal; DA, diacetyl; HEX, hexanal; HEXd12, hexanal deuterated; MDA malondialdehyde; GO, glyoxal.

Table S1. Precursor ion of the carbonyls derivatized with O-(2,3,4,5,6-pentafluorobenzyl)hydroxylamine hydrochloride (PFBHA), O-(tert-butyldimethylsilyl)hydroxylamine (TBS-ONH_2), hydroxylamine (NH_2OH), and methoxylamine hydrochloride (MOX).

**Carbonyl**	**PFBHA**		**TBS-ONH_2**		**NH_2OH**		**MOX**	
	Theorical	Observed	Theorical	Observed	Theorical	Observed	Theorical	Observed
2-butenal	263	265	199	184	85	-	99	-
Acetaldehyde	237	237	173	161	59	-	73	-
Acetone	251	252	187	182	73	-	87	-
Acrolein	249	266	185	160	71	-	85	-
Benzaldehyde	299	301	233	235	121	121	135	135
Deuterated acetone	257	259	193	189	79	-	93	-
Deuterated hexanal	303	307	239	226	115	-	129	140
Diacetyl	426	264	344	158	116	129	144	116
Formaldehyde	223	267	161	159	45	-	59	-
Furfural	289	257	225	225	111	-	125	-
Glyoxal	400	251	316	174	88	-	104	104
Hexanal	305	295	227	214	115	115	129	129
Malondialdehyde	412	250	328	196	102	102	130	117
Pentanal	291	281	213	200	101	101	115	115

Each compound was individually derivatized with the corresponding reagent and determined via GC-MS. Mass spectrums were record on scan mode at 70 eV in a mass range from 100-500 m/z.

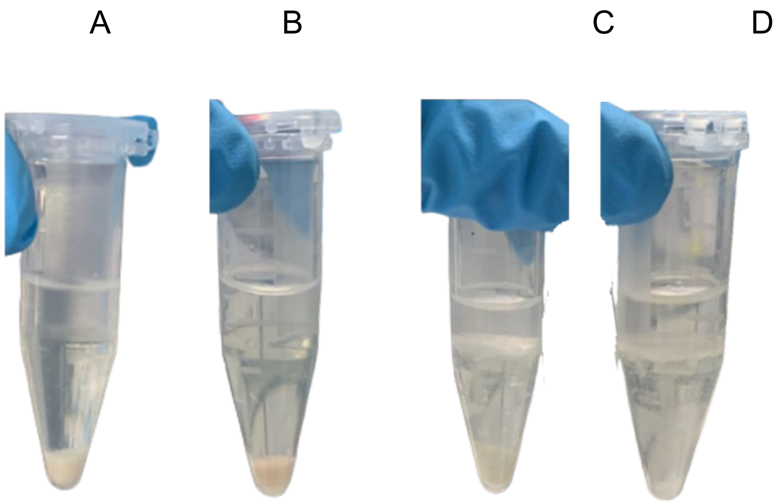

Figure S5. Protein precipitation by acetonitrile (A), isopropanol (B), methanol (C), ethanol (D).

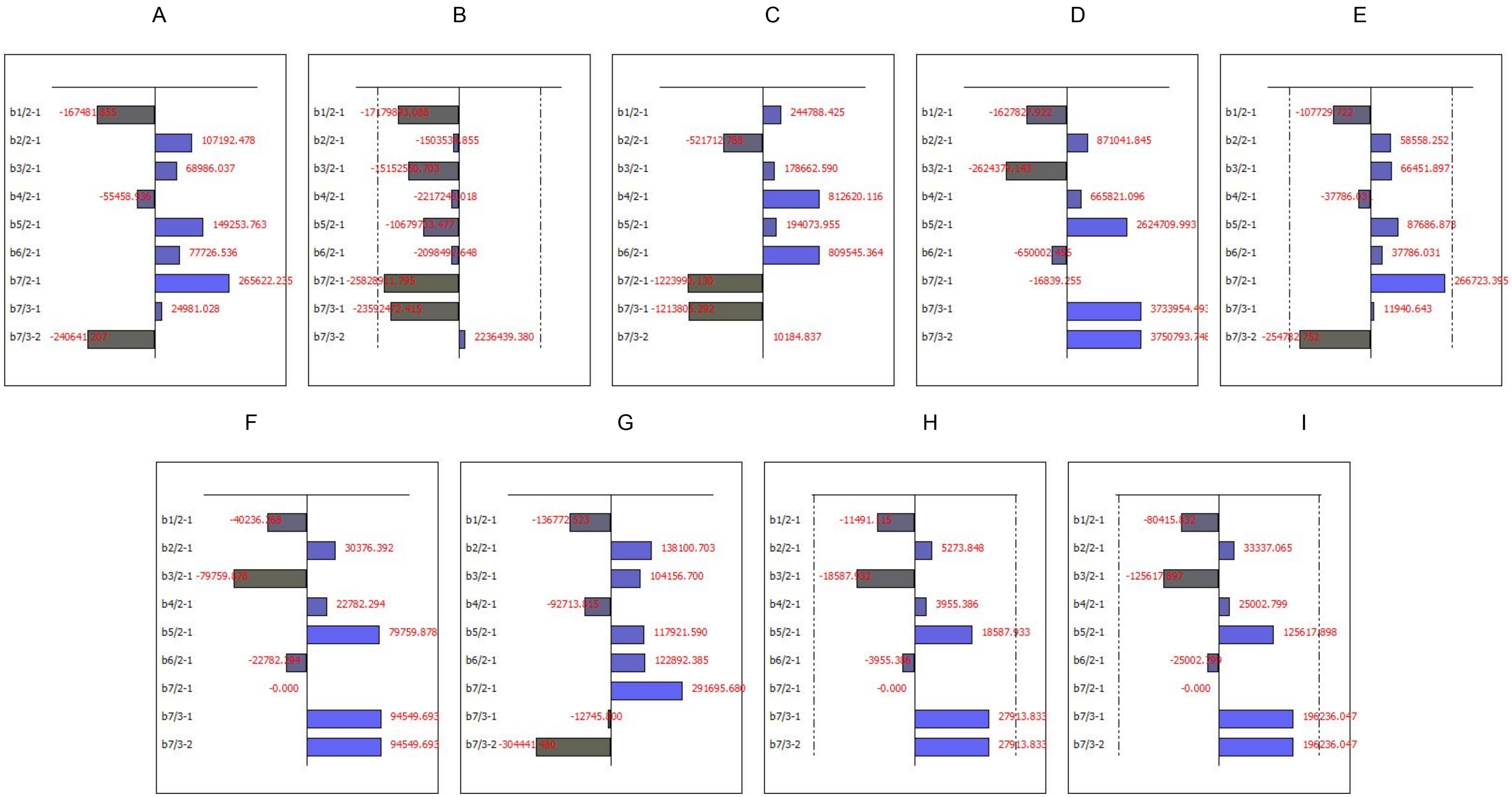

Figure S6. Delta weight plots from asymmetrical screening design. (A) 2-butenal, (B) acetaldehyde, (C) acetone, (D) deuterated acetone, (E) acrolein, (F) furfural, (G) hexanal, (H) malondialdehyde, (I) benzaldehyde

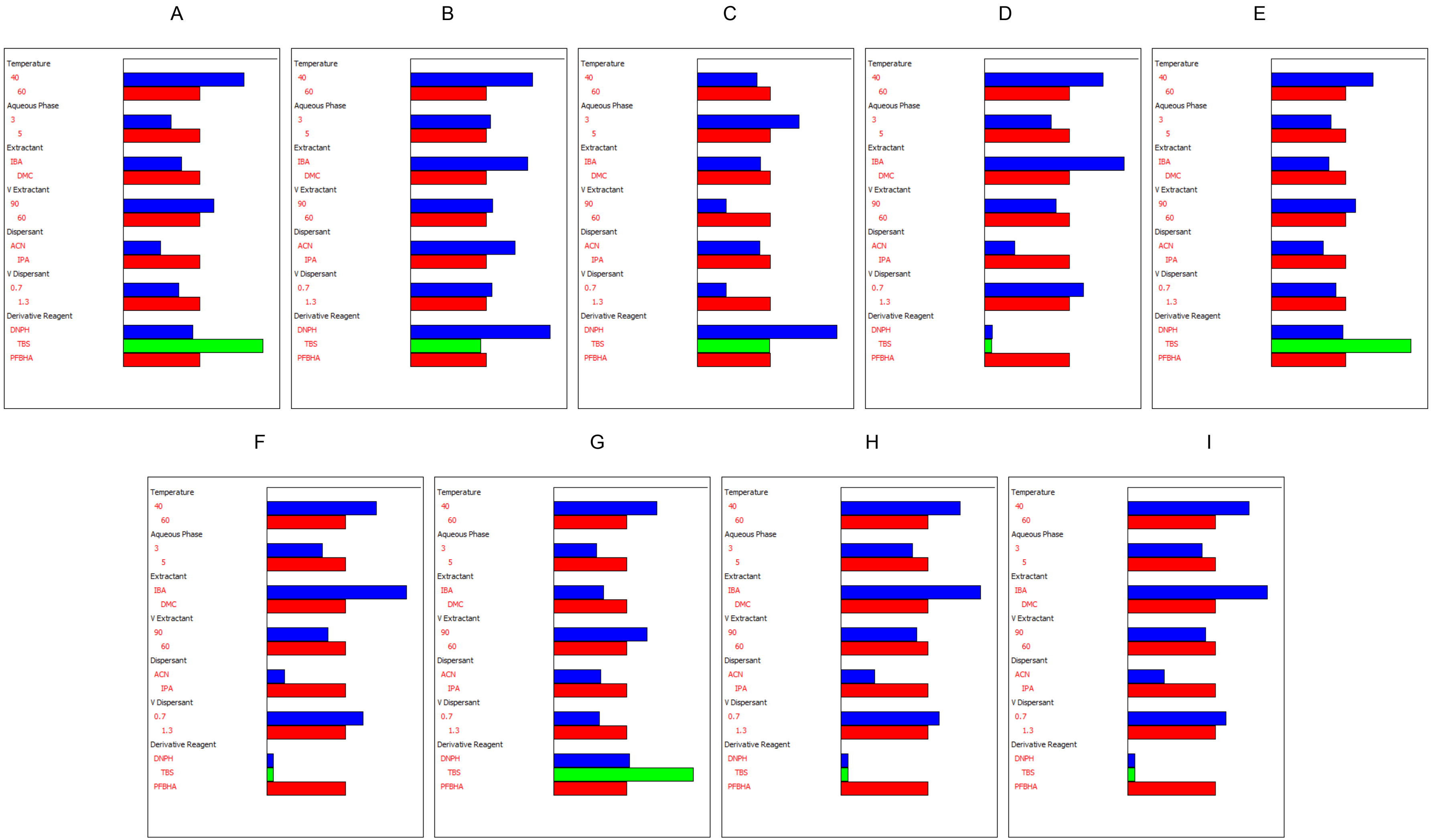

Figure S7. Total effects from asymmetrical screening design. (A) 2-butenal, (B) acetaldehyde, (C) acetone, (D) deuterated acetone, (E) acrolein, (F) furfural, (G) hexanal, (H) malondialdehyde, (I) benzaldehyde.

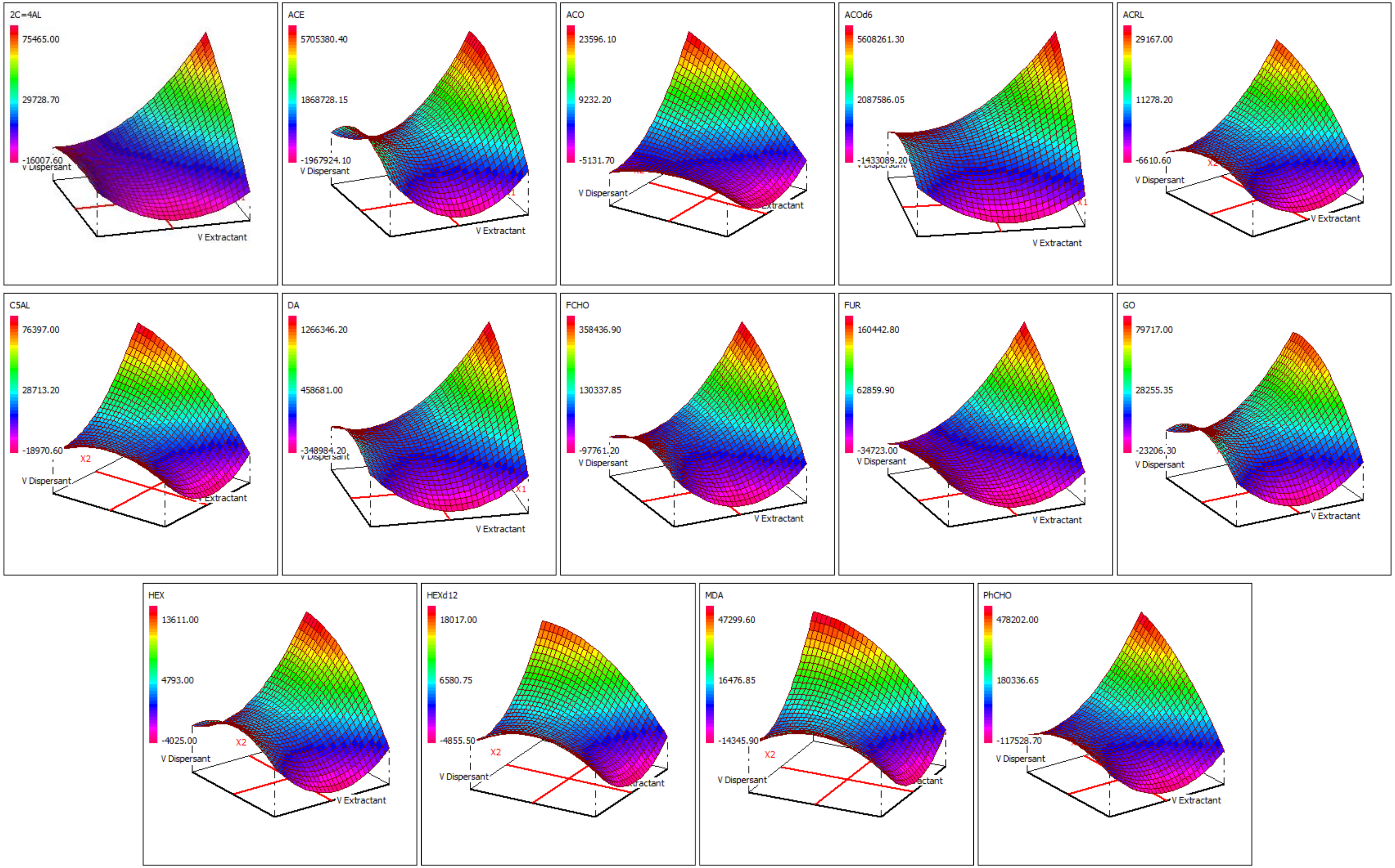

Figure S8. Surface Responses from central composite design. 2=C4AL, 2-butenal; ACE, acetaldehyde; ACO, acetone; ACOd6, deuterated acetone; ACRL, acrolein; C5AL, pentanal; DA, diacetyl; FCHO, formaldehyde; FUR, furfural; GO, glyoxal; HEX, hexanal; HEXd12, deuterated hexanal; MDA, malondialdehyde; PhCHO, benzaldehyde.

Table S2. Comparison of published dispersive liquid-liquid microextraction procedures for carbonyl compounds determination in food.

Analyte	Matrix	Agitation	Dispersant solvent	Extractant solvent	t min	T °C	Derivative reagent	Instrument	LOD ng/mL or ng/g	Recovery %	Ref.
FCHO	beverages	MW	ACN	IL 3453W	1.5	NR	DNPH	HPLC-UV	0.12	85-95	26
FCHO	Milk	flow-batch	MeOH	IL 3453W	0.75	45	ACAC	UV	100	91-103	43
FUR Hydroxymethylfurfural	Baby formula	manual	EtOH	1-octanol	2	NR	NR	HPLC-UV	0.7-1.8	92-98	44
GO	Alcoholic beverages	Vortex	1-butanol	CH_2Cl_2	15	NR	3,4-diaminopyridine	HPLC-FLD	1.5	NR	40
FCHO, ACE, C5AL, ACRL, MDA, Propanal	Vegetable oil	US	ACN	CH3Cl	5	60	DNPH	GC-MS	50-100	95-106	24
MDA, ACRL, 4-Hydroxy-2-nonenal	Beverages	US	ACN	CH3Cl	5	60	DNPH	GC-MS	50-200	94-102	32
FCHO, ACE, C5AL, HEX, Propanal, Butanal, Heptanal	Drinking water & alcoholic beverages	-	ACN	DES	10	40	DNPH	HPLC-UV	0.1-0.5	76-119	42
MDA, FCHO, ACE, ACRL, GO, DA, MGO, FUR, PhCHO	Coffee	US	EtOH	Isooctane	15	50	DNPH	GC-MS	271-435	90-108	41
ACE, FCHO, 2=C4AL, DA, ACO, C5AL, ACRL, FUR, HEX, PhCHO, GO, MDA	Plant-based beverages	US	IPA	IBA	20	40	PFBHA	GC-MS	8.5-272.9	90-107	This work

Ref. Reference; NR, non-reported; FCHO, formaldehyde; FUR, furfural; GO, glyoxal; ACE, acetaldehyde; C5AL, pentanal; ACRL, acrolein; MDA, malondialdehyde; HEX, hexanal; DA, diacetyl; MGO, methylglyoxal; PhCHO, benzaldehyde; 2=CAL, 2-butenal; ACO, acetone; MW, microwave; US, ultrasound; ACN, acetonitrile; MeOH, methanol; EtOH, ethanol; IPA, Isopropanol; IBA, isobutyl acetate; IL 3453W, ionic liquid Trihexyltetradecylphosphonium Chloride; DES, deep euthetic solvent; DNPH, 2,4-dinitrophenylhydrazine; ACAC, acetylacetone in an ammonium acetate (2.0% v/v); PFBHA, O-(2,3,4,5,6-pentafluorobenzyl)hydroxylamine hydrochloride; HPLC, high-performance liquid chromatography; UV, ultraviolet detector; FLD, flourenscense detector; GC, gas chromatography; MS, Mass spectrometry.

Table S3. Carbonyl occurrence in plant-based beverages.

PBB	Acetaldehyde		Formaldehyde		2-butenal		Diacetyl		Acetone		Pentanal		Acrolein		Furfural		Hexanal		Benzaldehyde		Glyoxal		Malondialdehyde	
	ng/mL	±σ	ng/mL	±σ	ng/mL	±σ	ng/mL	±σ	ng/mL	±σ	ng/mL	±σ	ng/mL	±σ	ng/mL	±σ	ng/mL	±σ	ng/mL	±σ	ng/mL	±σ	ng/mL	±σ
Al-1	734.8	31.2	<LOQ	-	<LOQ	-	1528.8	262.7	<LOQ	-	<LOQ	-	<LOQ	-	1536.1	304.1	360.3	0.6	<LOQ	-	252.0	30.6	<LOQ	-
Al-2	745.3	3.0	<LOQ	-	<LOQ	-	909.5	155.4	<LOQ	-	<LOQ	-	<LOQ	-	1229.2	73.6	355.3	3.2	<LOQ	-	183.6	9.0	<LOQ	-
Al-3	1213.1	63.9	<LOQ	-	<LOQ	-	1375.0	156.6	<LOQ	-	<LOQ	-	<LOQ	-	1073.4	62.4	352.1	0.3	<LOQ	-	333.6	44.0	<LOQ	-
Al-4	880.9	51.6	<LOQ	-	<LOQ	-	1533.5	312.3	<LOQ	-	<LOQ	-	<LOQ	-	1779.8	127.9	356.6	0.5	<LOQ	-	356.6	29.4	<LOQ	-
Al-5	<LOQ	-	<LOQ	-	<LOQ	-	756.9	72.0	<LOQ	-	<LOQ	-	<LOQ	-	2695.1	152.7	340.1	4.8	<LOQ	-	<LOQ	-	<LOQ	-
Al-6	479.0	155.0	1770.4	225.4	1056.4	564.3	1680.6	319.8	<LOQ	-	1508.5	453.6	1886.1	113.3	1268.8	190.4	295.9	1.2	321.2	101.8	1200.3	638.3	1772.6	48.1
Al-7	1661.9	314.4	1402.4	826.5	525.6	385.5	1756.2	627.3	<LOQ	-	1915.1	148.6	1663.9	345.2	1497.1	303.9	<LOQ	-	190.4	165.0	1003.3	436.4	1347.2	180.6
Al-8	1199.4	238.4	<LOQ	-	115.3	100.5	774.1	646.7	<LOQ	-	2284.2	799.5	<LOQ	-	1179.6	204.5	349.9	3.4	172.8	15.2	<LOQ	-	943.1	47.0
Al-9	1060.5	267.5	1021.6	179.8	201.5	56.4	516.4	181.5	<LOQ	-	1417.0	318.4	820.1	279.3	1239.7	209.0	297.2	11.9	1219.2	278.3	987.2	548.0	361.1	109.3
Al-10	1125.7	59.6	<LOQ	-	<LOQ	-	894.3	8.1	<LOQ	-	<LOQ	-	<LOQ	-	1167.7	98.0	354.3	4.5	<LOQ	-	323.8	3.6	<LOQ	-
R1	581.5	57.7	<LOQ	-	<LOQ	-	<LOQ	-	<LOQ	-	<LOQ	-	<LOQ	-	1295.2	594.4	351.7	12.8	<LOQ	-	225.9	28.2	<LOQ	-
R2	1470.0	32.2	<LOQ	-	<LOQ	-	1978.6	96.6	<LOQ	-	<LOQ	-	<LOQ	-	1996.7	98.3	349.7	3.2	<LOQ	-	796.4	51.1	<LOQ	-
R3	551.8	19.9	<LOQ	-	<LOQ	-	<LOQ	-	<LOQ	-	<LOQ	-	<LOQ	-	2011.8	31.8	349.9	13.0	<LOQ	-	177.4	9.6	<LOQ	-
R4	1396.3	34.9	<LOQ	-	<LOQ	-	1110.2	30.9	<LOQ	-	<LOQ	-	<LOQ	-	2179.6	265.4	351.7	1.0	<LOQ	-	757.2	100.1	<LOQ	-
R5	348.4	41.0	<LOQ	-	<LOQ	-	820.3	0.8	<LOQ	-	768.0	66.1	<LOQ	-	1620.6	199.1	331.7	8.5	<LOQ	-	<LOQ	-	<LOQ	-
R6	294.4	14.0	710.9	76.8	101.1	4.3	1636.0	31.0	<LOQ	-	236.2	61.8	<LOQ	-	1823.8	7.2	342.6	2.7	<LOQ	-	268.7	96.8	2404.6	134.2
R7	826.8	74.1	<LOQ	-	582.7	40.3	1398.9	178.7	<LOQ	-	456.9	69.7	<LOQ	-	1554.5	234.1	282.1	117.4	285.6	82.1	255.6	50.9	1661.3	56.5
O-1	911.9	10.0	<LOQ	-	50.2	3.1	722.4	70.1	<LOQ	-	<LOQ	-	<LOQ	-	1439.5	15.9	360.7	0.3	<LOQ	-	<LOQ	-	<LOQ	-
O-2	946.0	17.2	<LOQ	-	<LOQ	-	550.9	397.5	<LOQ	-	<LOQ	-	<LOQ	-	1560.2	98.7	360.4	0.6	<LOQ	-	<LOQ	-	<LOQ	-
O-3	955.1	14.5	<LOQ	-	<LOQ	-	933.7	40.1	<LOQ	-	<LOQ	-	<LOQ	-	1581.1	56.4	351.2	1.6	<LOQ	-	<LOQ	-	<LOQ	-
O-4	968.2	40.0	1533.0	132.1	<LOQ	-	680.6	23.5	<LOQ	-	<LOQ	-	<LOQ	-	1101.3	84.3	334.3	1.4	<LOQ	-	<LOQ	-	<LOQ	-
O-5	1514.2	46.4	<LOQ	-	<LOQ	-	<LOQ	-	<LOQ	-	280.1	23.5	<LOQ	-	1632.1	34.4	360.4	0.3	<LOQ	-	<LOQ	-	<LOQ	-
O-6	1318.0	67.1	<LOQ	-	117.6	4.9	1765.6	133.6	<LOQ	-	<LOQ	-	1762.0	234.3	1760.1	81.2	359.1	0.3	378.3	12.0	<LOQ	-	791.0	75.2
O-7	1091.4	59.4	<LOQ	-	<LOQ	-	1387.0	87.7	<LOQ	-	<LOQ	-	1037.8	79.8	1537.0	29.5	346.4	0.5	1424.0	423.3	<LOQ	-	<LOQ	-

O-8	1149.0	63.8	<LOQ	-	113.0	21.5	1629.6	75.4	<LOQ	-	<LOQ	-	1675.3	453.3	1269.5	275.5	340.4	3.7	311.9	69.9	<LOQ	-	<LOQ	-
O-9	1139.6	53.9	<LOQ	-	111.1	53.6	1548.5	422.9	<LOQ	-	210.6	17.7	<LOQ	-	1394.5	172.2	298.3	6.5	1207.5	14.0	<LOQ	-	1259.5	93.7
O-10	866.2	104.3	<LOQ	-	62.9	6.0	1506.5	397.8	<LOQ	-	<LOQ	-	930.8	48.4	1718.0	58.0	335.6	4.5	357.3	136.1	<LOQ	-	1209.1	328.4
O-11	705.8	18.9	<LOQ	-	<LOQ	-	758.0	13.7	<LOQ	-	1807.1	231.2	289.1	45.0	1552.7	195.2	328.3	8.9	<LOQ	-	<LOQ	-	<LOQ	-
O-12	1078.7	96.1	<LOQ	-	44.6	4.7	1231.0	19.9	<LOQ	-	<LOQ	-	1394.3	184.9	1844.7	16.8	358.8	0.3	5575.4	29.0	<LOQ	-	1255.7	113.0
O-13	881.4	12.3	<LOQ	-	<LOQ	-	973.3	20.9	<LOQ	-	<LOQ	-	<LOQ	-	1541.0	48.1	358.0	1.7	<LOQ	-	<LOQ	-	<LOQ	-
O-14	999.7	34.7	<LOQ	-	<LOQ	-	827.5	0.0	<LOQ	-	<LOQ	-	<LOQ	-	1654.1	62.4	350.0	1.1	<LOQ	-	<LOQ	-	<LOQ	-
S1	729.4	19.8	<LOQ	-	<LOQ	-	2449.5	11.9	<LOQ	-	<LOQ	-	<LOQ	-	2347.2	76.9	349.9	0.5	<LOQ	-	<LOQ	-	<LOQ	-
S2	509.8	12.7	<LOQ	-	<LOQ	-	1962.5	17.4	<LOQ	-	<LOQ	-	<LOQ	-	1684.6	66.5	356.7	0.4	<LOQ	-	<LOQ	-	<LOQ	-
S3	914.8	27.7	<LOQ	-	<LOQ	-	2473.3	262.0	<LOQ	-	<LOQ	-	<LOQ	-	2878.3	168.5	333.5	1.0	<LOQ	-	<LOQ	-	<LOQ	-
S4	703.5	35.0	<LOQ	-	<LOQ	-	2601.7	256.5	<LOQ	-	<LOQ	-	<LOQ	-	2875.6	587.4	337.8	7.3	<LOQ	-	<LOQ	-	<LOQ	-
S5	648.4	24.0	<LOQ	-	<LOQ	-	2264.2	176.9	<LOQ	-	<LOQ	-	<LOQ	-	2442.9	60.6	348.8	0.7	<LOQ	-	<LOQ	-	<LOQ	-
S6	537.6	19.3	<LOQ	-	<LOQ	-	701.7	40.6	<LOQ	-	1492.1	181.0	644.5	58.4	1395.0	318.5	318.4	11.2	<LOQ	-	<LOQ	-	<LOQ	-
S7	835.8	194.9	1096.9	23.7	420.4	79.0	2437.6	695.0	<LOQ	-	3994.2	740.8	6642.9	97.3	1166.7	109.8	<LOQ	-	285.4	56.4	355.1	40.9	2875.6	1877.9
S8	1385.3	321.9	659.8	69.5	482.6	17.2	2565.7	207.0	<LOQ	-	6299.7	240.8	13388.2	3306.4	1153.7	185.0	<LOQ	-	307.5	55.1	293.0	75.0	3039.3	365.0
C1	1040.7	11.3	<LOQ	-	<LOQ	-	<LOQ	-	<LOQ	-	<LOQ	-	<LOQ	-	2605.3	77.9	350.7	2.3	<LOQ	-	<LOQ	-	<LOQ	-
C2	<LOQ	-	<LOQ	-	<LOQ	-	783.8	30.0	<LOQ	-	505.7	57.9	<LOQ	-	2418.1	34.8	345.7	3.0	<LOQ	-	<LOQ	-	<LOQ	-
MC1	1236.5	12.3	<LOQ	-	<LOQ	-	1846.1	101.2	<LOQ	-	<LOQ	-	<LOQ	-	1913.4	56.8	536.1	14.6	<LOQ	-	<LOQ	-	<LOQ	-
MC2	789.9	5.1	<LOQ	-	<LOQ	-	1498.3	11.0	<LOQ	-	<LOQ	-	<LOQ	-	1705.6	63.8	687.2	29.7	<LOQ	-	<LOQ	-	<LOQ	-
MC3	486.1	32.9	<LOQ	-	<LOQ	-	1173.6	38.8	<LOQ	-	<LOQ	-	<LOQ	-	1567.0	58.5	593.3	60.9	<LOQ	-	<LOQ	-	<LOQ	-
M1	942.8	15.7	<LOQ	-	<LOQ	-	1467.8	128.2	<LOQ	-	<LOQ	-	<LOQ	-	1648.9	130.1	<LOQ	-	<LOQ	-	<LOQ	-	<LOQ	-
M2	905.3	13.9	<LOQ	-	<LOQ	-	1237.4	145.0	<LOQ	-	<LOQ	-	<LOQ	-	1777.8	121.3	<LOQ	-	<LOQ	-	505.8	18.7	<LOQ	-
M3	851.7	9.5	<LOQ	-	<LOQ	-	1346.1	48.7	<LOQ	-	<LOQ	-	<LOQ	-	2050.0	56.6	<LOQ	-	<LOQ	-	<LOQ	-	<LOQ	-
M4	989.8	12.3	<LOQ	-	<LOQ	-	1504.1	94.1	<LOQ	-	<LOQ	-	<LOQ	-	1487.0	99.2	<LOQ	-	<LOQ	-	<LOQ	-	<LOQ	-
M5	281.7	32.4	<LOQ	-	214.7	24.4	1755.1	100.3	<LOQ	-	725.6	51.2	1144.4	34.1	1338.9	653.2	<LOQ	-	236.3	23.5	180.4	25.1	1447.5	98.8
M6	1323.4	23.5	518.8	123.2	378.6	50.7	1458.8	210.2	<LOQ	-	446.3	100.6	649.3	88.2	469.6	130.8	<LOQ	-	170.8	16.3	308.5	19.4	1727.2	24.1
M7	1077.2	6.0	<LOQ	-	<LOQ	-	1530.1	137.6	<LOQ	-	<LOQ	-	<LOQ	-	1958.1	27.5	<LOQ	-	<LOQ	-	<LOQ	-	<LOQ	-

PBB, plant-based beverages; Al, almond; R, rice; O, oat; S, soybean; C, coconut; MC, minor crops (MC1, canary seed; MC2, rye; MC3, tiger-nut); M, mixed formulation (M1, oat–hazelnut–nut; M2, rice–almond; M3, soy–pea; M4, soy–oat–coconut; M5, rice–hazelnut; M6, oat–nut; M7, rice–coconut), <LOQ, below limit of quantification.

Table S4. Estimated intake after consumption of 1 glass of vegetal beverage (250mL) considering the average weight of general European population (75kg)

PBB	Acetaldehyde		Formaldehyde		2-butenal		Diacetyl		Acetone		Pentanal		Acrolein		Furfural		Hexanal		Benzaldehyde		Glyoxal		Malondialdehyde	
	ug/kg	±σ	ug/kg	±σ	ug/kg	±σ	ug/kg	±σ	ug/kg	±σ	ug/kg	±σ	ug/kg	±σ	ug/kg	±σ	ug/kg	±σ	ug/kg	±σ	ug/kg	±σ	ug/kg	±σ
Al-1	2.449	0.104	0.000	0.000	0.000	0.000	5.096	0.876	0.000	0.000	0.000	0.000	0.000	0.000	5.120	1.014	1.201	0.002	0.000	0.000	0.840	0.102	0.000	0.000
Al-2	2.484	0.010	0.000	0.000	0.000	0.000	3.032	0.518	0.000	0.000	0.000	0.000	0.000	0.000	4.097	0.245	1.184	0.011	0.000	0.000	0.612	0.030	0.000	0.000
Al-3	4.044	0.213	0.000	0.000	0.000	0.000	4.583	0.522	0.000	0.000	0.000	0.000	0.000	0.000	3.578	0.208	1.174	0.001	0.000	0.000	1.112	0.147	0.000	0.000
Al-4	2.936	0.172	0.000	0.000	0.000	0.000	5.112	1.041	0.000	0.000	0.000	0.000	0.000	0.000	5.933	0.426	1.189	0.002	0.000	0.000	1.189	0.098	0.000	0.000
Al-5	0.000	0.000	0.000	0.000	0.000	0.000	2.523	0.240	0.000	0.000	0.000	0.000	0.000	0.000	8.984	0.509	1.134	0.016	0.000	0.000	0.000	0.000	0.000	0.000
Al-6	1.597	0.517	5.901	0.751	3.521	1.881	5.602	1.066	0.000	0.000	5.028	1.512	6.287	0.378	4.229	0.635	0.986	0.004	1.071	0.339	4.001	2.128	5.909	0.160
Al-7	5.540	1.048	4.675	2.755	1.752	1.285	5.854	2.091	0.000	0.000	6.384	0.495	5.546	1.151	4.990	1.013	0.000	0.000	0.635	0.550	3.344	1.455	4.491	0.602
Al-8	3.998	0.795	0.000	0.000	0.384	0.335	2.580	2.156	0.000	0.000	7.614	2.665	0.000	0.000	3.932	0.682	1.166	0.011	0.576	0.051	0.000	0.000	3.144	0.157
Al-9	3.535	0.892	3.405	0.599	0.672	0.188	1.721	0.605	0.000	0.000	4.723	1.061	2.734	0.931	4.132	0.697	0.991	0.040	4.064	0.928	3.291	1.827	1.204	0.364
Al-10	3.752	0.199	0.000	0.000	0.000	0.000	2.981	0.027	0.000	0.000	0.000	0.000	0.000	0.000	3.892	0.327	1.181	0.015	0.000	0.000	1.079	0.012	0.000	0.000
R1	1.938	0.192	0.000	0.000	0.000	0.000	0.000	0.000	0.000	0.000	0.000	0.000	0.000	0.000	4.317	1.981	1.172	0.043	0.000	0.000	0.753	0.094	0.000	0.000
R2	4.900	0.107	0.000	0.000	0.000	0.000	6.595	0.322	0.000	0.000	0.000	0.000	0.000	0.000	6.656	0.328	1.166	0.011	0.000	0.000	2.655	0.170	0.000	0.000
R3	1.839	0.066	0.000	0.000	0.000	0.000	0.000	0.000	0.000	0.000	0.000	0.000	0.000	0.000	6.706	0.106	1.166	0.043	0.000	0.000	0.591	0.032	0.000	0.000
R4	4.654	0.116	0.000	0.000	0.000	0.000	3.701	0.103	0.000	0.000	0.000	0.000	0.000	0.000	7.265	0.885	1.172	0.003	0.000	0.000	2.524	0.334	0.000	0.000
R5	1.161	0.137	0.000	0.000	0.000	0.000	2.734	0.003	0.000	0.000	2.560	0.220	0.000	0.000	5.402	0.664	1.106	0.028	0.000	0.000	0.000	0.000	0.000	0.000
R6	0.981	0.047	2.370	0.256	0.337	0.014	5.453	0.103	0.000	0.000	0.787	0.206	0.000	0.000	6.079	0.024	1.142	0.009	0.000	0.000	0.896	0.323	8.015	0.447
R7	2.756	0.247	0.000	0.000	1.942	0.134	4.663	0.596	0.000	0.000	1.523	0.232	0.000	0.000	5.182	0.780	0.940	0.391	0.952	0.274	0.852	0.170	5.538	0.188
O-1	3.040	0.033	0.000	0.000	0.167	0.010	2.408	0.234	0.000	0.000	0.000	0.000	0.000	0.000	4.798	0.053	1.202	0.001	0.000	0.000	0.000	0.000	0.000	0.000
O-2	3.153	0.057	0.000	0.000	0.000	0.000	1.836	1.325	0.000	0.000	0.000	0.000	0.000	0.000	5.201	0.329	1.201	0.002	0.000	0.000	0.000	0.000	0.000	0.000
O-3	3.184	0.048	0.000	0.000	0.000	0.000	3.112	0.134	0.000	0.000	0.000	0.000	0.000	0.000	5.270	0.188	1.171	0.005	0.000	0.000	0.000	0.000	0.000	0.000
O-4	3.227	0.133	5.110	0.440	0.000	0.000	2.269	0.078	0.000	0.000	0.000	0.000	0.000	0.000	3.671	0.281	1.114	0.005	0.000	0.000	0.000	0.000	0.000	0.000
O-5	5.047	0.155	0.000	0.000	0.000	0.000	0.000	0.000	0.000	0.000	0.934	0.078	0.000	0.000	5.440	0.115	1.201	0.001	0.000	0.000	0.000	0.000	0.000	0.000
O-6	4.393	0.224	0.000	0.000	0.392	0.016	5.885	0.445	0.000	0.000	0.000	0.000	5.873	0.781	5.867	0.271	1.197	0.001	1.261	0.040	0.000	0.000	2.637	0.251

O-7	3.638	0.198	0.000	0.000	0.000	0.000	4.623	0.292	0.000	0.000	0.000	0.000	3.459	0.266	5.123	0.098	1.155	0.002	4.747	1.411	0.000	0.000	0.000	0.000
O-8	3.830	0.213	0.000	0.000	0.377	0.072	5.432	0.251	0.000	0.000	0.000	0.000	5.584	1.511	4.232	0.918	1.135	0.012	1.040	0.233	0.000	0.000	0.000	0.000
O-9	3.799	0.180	0.000	0.000	0.370	0.179	5.162	1.410	0.000	0.000	0.702	0.059	0.000	0.000	4.648	0.574	0.994	0.022	4.025	0.047	0.000	0.000	4.198	0.312
O-10	2.887	0.348	0.000	0.000	0.210	0.020	5.022	1.326	0.000	0.000	0.000	0.000	3.103	0.161	5.727	0.193	1.119	0.015	1.191	0.454	0.000	0.000	4.030	1.095
O-11	2.353	0.063	0.000	0.000	0.000	0.000	2.527	0.046	0.000	0.000	6.024	0.771	0.964	0.150	5.176	0.651	1.094	0.030	0.000	0.000	0.000	0.000	0.000	0.000
O-12	3.596	0.320	0.000	0.000	0.149	0.016	4.103	0.066	0.000	0.000	0.000	0.000	4.648	0.616	6.149	0.056	1.196	0.001	18.585	0.097	0.000	0.000	4.186	0.377
O-13	2.938	0.041	0.000	0.000	0.000	0.000	3.244	0.070	0.000	0.000	0.000	0.000	0.000	0.000	5.137	0.160	1.193	0.006	0.000	0.000	0.000	0.000	0.000	0.000
O-14	3.332	0.116	0.000	0.000	0.000	0.000	2.758	0.000	0.000	0.000	0.000	0.000	0.000	0.000	5.514	0.208	1.167	0.004	0.000	0.000	0.000	0.000	0.000	0.000
S1	2.431	0.066	0.000	0.000	0.000	0.000	8.165	0.040	0.000	0.000	0.000	0.000	0.000	0.000	7.824	0.256	1.166	0.002	0.000	0.000	0.000	0.000	0.000	0.000
S2	1.699	0.042	0.000	0.000	0.000	0.000	6.542	0.058	0.000	0.000	0.000	0.000	0.000	0.000	5.615	0.222	1.189	0.001	0.000	0.000	0.000	0.000	0.000	0.000
S3	3.049	0.092	0.000	0.000	0.000	0.000	8.244	0.873	0.000	0.000	0.000	0.000	0.000	0.000	9.594	0.562	1.112	0.003	0.000	0.000	0.000	0.000	0.000	0.000
S4	2.345	0.117	0.000	0.000	0.000	0.000	8.672	0.855	0.000	0.000	0.000	0.000	0.000	0.000	9.585	1.958	1.126	0.024	0.000	0.000	0.000	0.000	0.000	0.000
S5	2.161	0.080	0.000	0.000	0.000	0.000	7.547	0.590	0.000	0.000	0.000	0.000	0.000	0.000	8.143	0.202	1.163	0.002	0.000	0.000	0.000	0.000	0.000	0.000
S6	1.792	0.064	0.000	0.000	0.000	0.000	2.339	0.135	0.000	0.000	4.974	0.603	2.148	0.195	4.650	1.062	1.061	0.037	0.000	0.000	0.000	0.000	0.000	0.000
S7	2.786	0.650	3.656	0.079	1.401	0.263	8.125	2.317	0.000	0.000	13.314	2.469	22.143	0.324	3.889	0.366	0.000	0.000	0.951	0.188	1.184	0.136	9.585	6.260
S8	4.618	1.073	2.199	0.232	1.609	0.057	8.552	0.690	0.000	0.000	20.999	0.803	44.627	11.021	3.846	0.617	0.000	0.000	1.025	0.184	0.977	0.250	10.131	1.217
C1	3.469	0.038	0.000	0.000	0.000	0.000	0.000	0.000	0.000	0.000	0.000	0.000	0.000	0.000	8.684	0.260	1.169	0.008	0.000	0.000	0.000	0.000	0.000	0.000
C2	0.000	0.000	0.000	0.000	0.000	0.000	2.613	0.100	0.000	0.000	1.686	0.193	0.000	0.000	8.060	0.116	1.152	0.010	0.000	0.000	0.000	0.000	0.000	0.000
MC1	4.122	0.041	0.000	0.000	0.000	0.000	6.154	0.337	0.000	0.000	0.000	0.000	0.000	0.000	6.378	0.189	1.787	0.049	0.000	0.000	0.000	0.000	0.000	0.000
MC2	2.633	0.017	0.000	0.000	0.000	0.000	4.994	0.037	0.000	0.000	0.000	0.000	0.000	0.000	5.685	0.213	2.291	0.099	0.000	0.000	0.000	0.000	0.000	0.000
MC3	1.620	0.110	0.000	0.000	0.000	0.000	3.912	0.129	0.000	0.000	0.000	0.000	0.000	0.000	5.223	0.195	1.978	0.203	0.000	0.000	0.000	0.000	0.000	0.000
M1	3.143	0.052	0.000	0.000	0.000	0.000	4.893	0.427	0.000	0.000	0.000	0.000	0.000	0.000	5.496	0.434	0.000	0.000	0.000	0.000	0.000	0.000	0.000	0.000
M2	3.018	0.046	0.000	0.000	0.000	0.000	4.125	0.483	0.000	0.000	0.000	0.000	0.000	0.000	5.926	0.404	0.000	0.000	0.000	0.000	1.686	0.062	0.000	0.000
M3	2.839	0.032	0.000	0.000	0.000	0.000	4.487	0.162	0.000	0.000	0.000	0.000	0.000	0.000	6.833	0.189	0.000	0.000	0.000	0.000	0.000	0.000	0.000	0.000
M4	3.299	0.041	0.000	0.000	0.000	0.000	5.014	0.314	0.000	0.000	0.000	0.000	0.000	0.000	4.957	0.331	0.000	0.000	0.000	0.000	0.000	0.000	0.000	0.000
M5	0.939	0.108	0.000	0.000	0.716	0.081	5.850	0.334	0.000	0.000	2.419	0.171	3.815	0.114	4.463	2.177	0.000	0.000	0.788	0.078	0.601	0.084	4.825	0.329
M6	4.411	0.078	1.729	0.411	1.262	0.169	4.863	0.701	0.000	0.000	1.488	0.335	2.164	0.294	1.565	0.436	0.000	0.000	0.569	0.054	1.028	0.065	5.757	0.080

M7	3.591	0.020	0.000	0.000	0.000	0.000	5.100	0.459	0.000	0.000	0.000	0.000	0.000	0.000	6.527	0.092	0.000	0.000	0.000	0.000	0.000	0.000	0.000	0.000

PBB, plant-based beverages; Al, almond; R, rice; O, oat; S, soybean; C, coconut; MC, minor crops (MC1, canary seed; MC2, rye; MC3, tiger-nut); M, mixed formulation (M1, oat–hazelnut–nut; M2, rice–almond; M3, soy–pea; M4, soy–oat–coconut; M5, rice–hazelnut; M6, oat–nut; M7, rice–coconut), <LOQ, below limit of quantification.
